# Identification and characterization of stromal-like cells with CD207^+/low^ CD1a^+/low^ phenotype derived from histiocytic lesions – a perspective in vitro model for drug testing

**DOI:** 10.1186/s12885-023-11807-0

**Published:** 2024-02-12

**Authors:** Agnieszka Śmieszek, Klaudia Marcinkowska, Zofia Małas, Mateusz Sikora, Martyna Kępska, Beata A. Nowakowska, Marta Deperas, Marta Smyk, Carlos Rodriguez-Galindo, Anna Raciborska

**Affiliations:** 1 Department of Pharmacology and Toxicology, Faculty of Veterinary Medicine, Wroclaw University of Environmental and Life Sciences, Norwida 31, 50-375 Wroclaw, Poland; 2 Department of Experimental Biology, Faculty of Biology and Animal Science, Wroclaw University of Environmental and Life Sciences, Norwida 27B, 50-375 Wroclaw, Poland; 3 Department of Oncology and Surgical Oncology for Children and Youth, Institute of Mother and Child, Kasprzaka 17a, 01-211 Warsaw, Poland; 4 Medical Genetics Department, Cytogenetics Laboratory, Institute of Mother and Child, Kasprzaka 17a, 01-211 Warsaw, Poland; 5 Jude Children’s Research Hospital, 262 Danny Thomas Place, Memphis, TN 38105 USA

**Keywords:** Histiocytoses, Langerhans cell histiocytosis, Rare disorders, Cell lines, In vitro study, Cellular model

## Abstract

**Background:**

Histiocytoses are rare disorders manifested by increased proliferation of pathogenic myeloid cells sharing histological features with macrophages or dendritic cells and accumulating in various organs, i.a., bone and skin. Pre-clinical in vitro models that could be used to determine molecular pathways of the disease are limited, hence research on histiocytoses is challenging. The current study compares cytophysiological features of progenitor, stromal-like cells derived from histiocytic lesions (sl-pHCs) of three pediatric patients with different histiocytoses types and outcomes. The characterized cells may find potential applications in drug testing.

**Methods:**

Molecular phenotype of the cells, i.e. expression of CD1a and CD207 (langerin), was determined using flow cytometry. Cytogenetic analysis included GTG-banded metaphases and microarray (aCGH) evaluation. Furthermore, the morphology and ultrastructure of cells were evaluated using a confocal and scanning electron microscope. The microphotographs from the confocal imaging were used to reconstruct the mitochondrial network and its morphology. Basic cytophysiological parameters, such as viability, mitochondrial activity, and proliferation, were analyzed using multiple cellular assays, including Annexin V/7-AAD staining, mitopotential analysis, BrdU test, clonogenicity analysis, and distribution of cells within the cell cycle. Biomarkers potentially associated with histiocytoses progression were determined using RT-qPCR at mRNA, miRNA and lncRNA levels. Intracellular accumulation of histiocytosis-specific proteins was detected with Western blot. Cytotoxicyty and IC50 of vemurafenib and trametinib were determined with MTS assay.

**Results:**

Obtained cellular models, i.e. RAB-1, HAN-1, and CHR-1, are heterogenic in terms of molecular phenotype and morphology. The cells express CD1a/CD207 markers characteristic for dendritic cells, but also show intracellular accumulation of markers characteristic for cells of mesenchymal origin, i.e. vimentin (VIM) and osteopontin (OPN). In subsequent cultures, cells remain viable and metabolically active, and the mitochondrial network is well developed, with some distinctive morphotypes noted in each cell line. Cell-specific transcriptome profile was noted, providing information on potential new biomarkers (non-coding RNAs) with diagnostic and prognostic features. The cells showed different sensitivity to vemurafenib and trametinib.

**Conclusion:**

Obtained and characterized cellular models of stromal-like cells derived from histiocytic lesions can be used for studies on histiocytosis biology and drug testing.

**Supplementary Information:**

The online version contains supplementary material available at 10.1186/s12885-023-11807-0.

## Introduction

Histiocytoses are a group of rare disorders characterized by the abnormal proliferation of macrophages (histiocytes), dendritic cells, or monocyte-derived cells, which accumulate in various tissues and organs. Those cells can infiltrate nearly any anatomical site, although they exhibit a predilection for specific tissues and organs, including the skin, bone, lung, lymph nodes, central nervous system, and heart [1, 2].

Histiocytoses mainly affect pediatric patients but can also occur in adults, ranging from benign self-limiting lesions to disseminated life-threatening cases. The phenotypic and clinical heterogeneity of histiocytoses is reflected in the over 100 subtypes, each with distinct clinical manifestations and histopathological characteristics [3]. Moreover, the histiocytes population within the lesion exhibits marked heterogeneity, while the histopathological and phenotypic features of certain types of histiocytosis may be challenging to differentiate [1, 4].

Considering those difficulties, in 2016, the Histiocyte Society (HS) provided a revised classification system to unify the histiocytoses grouping, simultaneously endorsing specific guidelines and recommendations for diagnoses of these rare disorders [3]. The classification system categorizes histiocytoses into five groups based on their clinical, histological, and molecular features [1, 3]. As a consequence, we can distinguish histiocytoses classified in the following groups, i.e. (L-group) Langerhans-related, (C-group) cutaneous and mucocutaneous, (M group) malignant histiocytoses, and (R-group) Rosai-Dorfman disease, as well as (H group) hemophagocytic lymphohistiocytosis and macrophage activation syndrome [1, 2, 5].

Although histiocytosis studies face several difficulties resulting from these diseases’ rarity, we are witnessing notable advancements in this field. The studies in the realm of histiocytoses aim to identify somatic clonal mutations and unique biomarkers to understand the molecular cause underlying the development of the disease. Such efforts are essential in facilitating the proper identification of uniform or overlapping entities and help determine the most effective preventive or therapeutic strategies. This approach also requires studies using in vitro model as an essential tool for exploring the biology and pathogenesis of histiocytoses and the cellular mechanisms underlying the disease process.

Several primary cell lines derived mainly from LCH lesions were characterized by their immunophenotype, cytokine production, and differentiation. The LCH lesion is a mixture of various cells, and the stromal-like cell lineage was also identified in this tissue microenvironment. The stromal-like cells were considered a population involved in the initiation and progression of LCH. However, this thread was not pursued further. It was found that the stromal-like cells found in tissues affected by histiocytosis shared the phenotype characteristic for Langerhans cells (LCs), i.e. expression of langerin (CD207) and CD1a molecule. The other specific phenotypic identifier was not found, even though the S100 marker was considered but ultimately recognized as non-specific.

Furthermore, Gogusev et al., in 2005, introduced the DOR-1, i.e. an LCH-derived cell line from a progressive LCH lesion of bone. The DOR-1 cells were described as expressing CD10^+^ and CD117^+^ surface biomarkers, as well as vimentin (VIM). However, the cells did not show expression of LC-specific markers, i.e. CD207 and CD1a. Under in vitro conditions, DOR-1 cells manifested the morphology of fibroblast-like cells, with elongated spindle-shaped cells expressing the potential to differentiate into a mature stromal-like lineage [6]. Moreover, the same scientific team characterized the PRU-1 cell line, also established from LCH bone lesion, but possessing stromal dermal dendritic cell (DDC) features and showing high cytokeratin expression [7].

The cell lines obtained from LCH lesions were considered a potential model for drug testing and development, but also a model allowing exploring the cellular mechanisms underlying the disease process [6, 7]. Considering those arguments, in the first non-commercial clinical trial, i.e. POLHISTIO, we have included much space for the strictly scientific task of obtaining primary cell lines from lesions affected by histiocytosis.

**Fig. 1:**
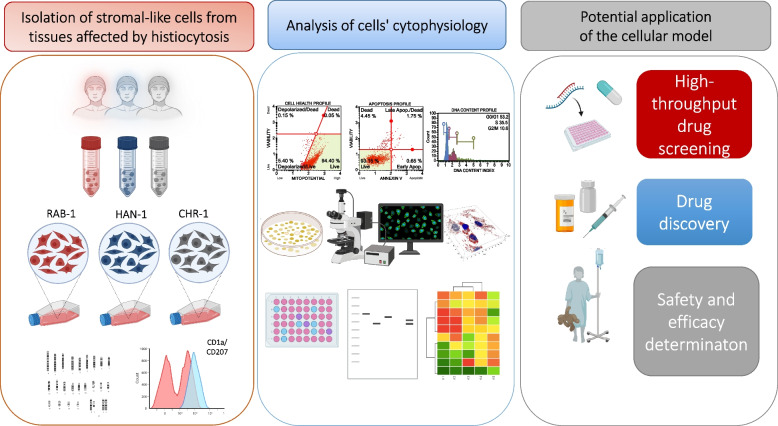
The experimental design and aims of the research. Three primary cell lines were obtained from patients with histiocytoses, i.e. RAB-1, HAN-1 and CHR-1. The cells share cytophysiological features of stromal cells with the phenotype of histiocytes progenitors (sl-pHCs). Their proliferative and metabolic activity was determined, including profiling the expression of transcripts and proteins associated with histiocytoses progression. The sl-pHCs were also used to test the cytotoxicity of vemurafenib and trametinib. Obtained and characterized cell lines present a valuable tool for further studies for

In this study, we have established, characterized, and compared three primary cell lines with low expression of CD207 and CD1a markers. The cells were isolated from tissues affected by histiocytic lesions and derived from LCH skin and bone (RAB-1 and CHR-1 cell lines, respectively) and non-LCH patient’s skin (HAN-1 cell line). We have evaluated those cell lines’ phenotypic features and basic cytophysiology, including morphology, ultrastructure, proliferation potential, and viability. The analysis included evaluating levels of both coding and non-coding RNAs present within the cells. We have also emphasized some distinctive features of obtained cells, such as mitochondrial morphology as well as metabolic activity. Additionally, we have determined levels of transcripts potentially associated with histiocytoses development and progression. Finally, we have determined the response of cells to inhibitors of B-Raf protein (BRAF) and mitogen-activated protein kinase kinase (MEK), i.e. vemurafenib and trametinib. The general overview of our study s presented in Fig. 1, which also indicates the possible future perspectives resulting from this research.

The morphology of cells, immunophenotype, and expression profile of genes and proteins indicate that obtained cells resemble stromal cells and thus were termed stromal-like precursors of histiocytes (sl-pHCs).

## Materials and methods

### Patients, cell isolation and culture of stromal-like progenitors of histiocytes (sl-pHCs)

The histiocytes derived from stromal tissue (sl-pHCs) were isolated from the histiocytosis lesions of three patients. The detailed characterization of patients is presented in Table 1. Histological analysis of biopsies was performed to confirm histiocytosis type. This study was conducted following the Declaration of Helsinki after approval by the Local Institutional Review Board at The Institute of Mother and Child (no. 28/2022). Moreover, written consent to use the biopsy material for cell isolation was obtained from the patient’s parents. Biopsies were collected at surgery, and the tissue fragments were transported in Hanks’ Balanced Salt Solution (HBSS) supplemented with 1% penicillin/streptomycin mix (P/S). The collected tissue samples (approximately 0.5 cm^2^) were transported to the cell culture laboratory within 24 h.

**Table 1:** Patient’s clinical characteristics

Patient code in the clinical study	Gender/Age	Diagnosis	Histiocytosis type	Treatment	Mutation in the pathological tissue	Mutation in the blood	Outcome	Name of the cell line
002	F/5y	LCH	Multisystem (skin, bone) R0 plus; relapse, neurodegenerative disease	CHT, TKI	BRAF	BRAF	AWD	RAB-1
014	F/1y	Non-LCH	Single sytem (skin)	None	None	None	CR	HAN-1
007	M/17y	LCH	Single system (bone), multifocal, R0 minus; relapse	CHT	BRAF	None	CR	CHR-1

*F* female, *M* male, *LCH* Langerhans cell histiocytosis, *R0* risk organ, *CHT* chemotherapy, *TKI* tyrosine kinase inhibitor, *AWD* alive with disease, *CR* complete remission

Isolation of cells was performed in aseptic conditions, with a method established previously for stromal cells [8]. The biopsies were cut into smaller fragments and placed into collagenase type I (1 mg/mL). The solution was prepared in Dulbecco’s modified Eagle’s medium/F12 (DMEM/F12). Obtained tissue samples were incubated with enzyme for 40 min in a CO_2_ incubator at 37 °C with periodic vortexing. After digestion, the suspension was centrifuged at 300 × g for 10 min. Homogenates were filtered using a sterile 70-μm cell strainer to remove undigested tissue fragments. The obtained suspension was washed with HBSS and centrifuged once again at 300 × g for 10 min. The cell pellets were suspended in a complete growth medium (CGM) consisting of low glucose Dulbecco's Modified Eagle Medium (LG DMEM) supplemented with 10% of fetal bovine serum (FBS) and 1% P/S solution. Primary cultures were transferred into culture flasks with a growth area of 12.5 cm^2^. After reaching 80% confluency, the cultures were passaged using StableCell™ Trypsin solution. To improve cells’ expansion, cultures were subsequently maintained in flasks with a growth surface area of 25 and 75 cm^2^. The primary and subsequent cultures were cultured at constant conditions in a CO_2_ incubator at 37 °C and maintained 95% humidity. Cytophysiology of sl-pHCs was compared in cultures at passage 5 (p5).

All reagents used in this procedure were derived from Merck (Poznan, Poland), while plastic materials were obtained from JET Biofil (Alchem, Torun, Poland).

### Conventional karyotype analysis

Both conventional karyotypes on GTG-banded metaphases and microarray (aCGH) analyses were performed on cells at the same passage (p6). Mitotic cells were collected after overnight incubation with 0.1 μg/ml of colcemid (Gibco,KaryoMAX™). Cell cultures were detached with trypsin solution. The obtained suspension was transferred to 15 ml tubes (Sarstedt, USA), centrifuged, and incubated with hypotonic solution (0.075M KCL). The fixation procedure was performed according to the standard protocol [6].

### Array comparative genomic hybridization (aCGH)

DNA from sl-pHCs cell lines was extracted using the Sherlock AX DNA isolation kit (A&A Biotechnology, Gdansk, Poland), according to the manufacturer’s instructions. Array comparative genomic hybridization (aCGH) was performed using the 60K CytoSure Constitutional v3 microarray (Oxford Gene Technology, Oxford, UK), according to the manufacturer’s protocol. Quality control measures were monitored using CytoSure Interpret Software (Oxford Gene Technology). Data analysis was performed using CytoSure Interpret Software (Oxford Gene Technology, Oxford, UK) and a circular binary segmentation algorithm. The CNVs were classified using the CytoSure Interpret Software (Oxford Gene Technology, Oxford, UK). The microarray used in this analysis does not contain SNP probes nor detect polyploidy, balanced chromosomal rearrangements, and loss of heterozygosity.

### Analysis of CD207 and CD1a immunophenotype in sl-pHCs cultures

Single antibody stains were performed on cells (1 × 10^6^) prepared in phosphate-buffered saline (PBS) with 5% FBS. The sl-pHCs were stained against CD207 (langerin) and CD1a. The stained cells were incubated with mouse monoclonal anti-human CD207 (MA5-38473, 1:200; Thermofisher Scientific, Warsaw, Poland) or CD1a (ab26935, 1 ug/ml; Abcam, Cambridge, UK) at room temperature for 30 min in the dark. Additionally, prior CD207 staining cells were permeabilized with a detergent-based permeabilizing agent for 30 min at room temperature. Cells were then incubated with a goat anti-mouse cross-adsorbed secondary antibody, Alexa Fluor™ 700 (A-21036, 10 ug/ml; Thermofisher Scientific, Warsaw, Poland). After each step of the staining protocol, the cells were washed and resuspended in 500 µl of PBS. The measurements were performed using BD LSR Fortessa (Becton–Dickinson and Company, Franklin Lakes, NJ, USA). The cells were gated and analyzed using BD FACSDiva™ (BD Biosciences v6.0). At least 100,000 cells were acquired for analysis. Unstained cells served as a negative control.

### Morphology and ultrastructure of sl-pHCs

For the analysis, sl-pHCs were seeded at a density equal 1.5 × 10^4^ on a 24-well culture plate coated with cover glass. The morphology, growth pattern, and ultrastructure of sl-pHCs cultures were analyzed based on microphotographs obtained with a confocal microscope (Leica TCS SPE, Leica Microsystems, KAWA.SKA Sp. z o.o., Zalesie Gorne, Poland) and scanning electron microscope (SEM, Evo LS 15, Zeiss, Oberkochen, Germany). The changes in the morphology of sl-pHCs cultures were monitored after 48, 96- and 168h of propagation. Cultures of sl-pHCs were stained to visualize nuclei, cytoskeleton, and mitochondrial network applying an established protocol published previously [8, 9].

The observations were made under a confocal microscope using magnifications 630 × and 1000 ×. Microphotographs were further processed with Fiji software (ImageJ 1.52n, Wayne Rasband, National Institute of Health, Bethesda, MD, USA). Additionally, three-dimensional (3D) reconstructions of the mitochondrial network complexity were performed using Leica Application Suite X (version 3.5.2.18963, Leica Microsystems CMS GmbH). MicroP software (ver. 1.1.11b, Biomedical Image Informatics Lab, Taipei City, Taiwan (R.O.C.) Institute of Biomedical Informatics, National Yang-Ming Chiao Tung University) powered by MATLAB (version R2010b, The MathWorks, Natick, MA, USA) was used to determine mitochondrial morphology.

The preparation of cultures for SEM analysis was also performed using the previously established method described in detail elsewhere [9]. SEM microphotographs were captured at magnifications: 500 × , 1000 × , and 2000 ×.

### Evaluation of cells viability and mitochondrial potential

The viability of subconfluent sl-pHCs was determined by analysing the apoptosis profile and mitochondrial membrane potential. The viability was established in the test with Muse® Annexin V and Dead Cell Kit, while mitochondrial activity was evaluated using Muse® Mitopotential Assay Kit. For each analysis, 1 × 10^4^ cells were used. The assays were performed following protocols provided by the manufacturer (Luminex/Merck, Poznan, Poland). The protocols were also fully described previously [9, 10]. Moreover, the metabolic activity of sl-pHCs was monitored using an MTS assay (Abcam, Cambridge, UK). The test was performed on semiconfluent sl-pHCs, and the metabolism was determined after 48, 96, and 168h of cells’ propagation. Cells were seeded at a density equal to 2.5 × 10^3^ on a 96-well plate and were cultured in 200 μL of CGM. The metabolic activity of cells was determined by adding 20 μL of MTS solution per well, followed by incubation with the dye for 2 h at 37 °C in a CO_2_ incubator. After that, absorbance was measured spectrophotometrically with a plate reader (Epoch Biotek, Biokom, Janki, Poland) at a wavelength of 490 nm. Acquired results were background corrected and analyzed.

### Evaluation of the proliferative potential of cell lines

The proliferative activity of subconfluent sl-pHCs was determined with regard to distribution in the cell cycle and population doubling time (PDT). The cells were stained with Muse® Cell Cycle Kit and Muse® Count and Viability Kit, respectively. The analysis was performed on Muse Cell Analyzer – the reagents and analyser derived from Luminex/Merck (Poznan, Poland). The number of cells was determined routinely, and obtained data were used to calculate PDT with an algorithm published by Heuer et al. [11] and Cell Calculator [12]. The proliferation of sl-pHCs was also evaluated based on the BrdU incorporation assay (Abcam, Symbios, Straszyn, Polska). For BrdU analysis, cells were seeded at density 2.5 × 10^3^on a 96-well plate. All tests were performed accordingly to well-described methods [8, 13] and in compliance with protocols provided by the manufacturers of the assays. Furthermore, colony-forming unit (CFU-E) occurrence efficiency was assessed after ten days of culture using the earlier protocol for stromal [8] and cancer cells [14].

### Analysis of the transcriptome

The total RNA was isolated from sl-pHCs using the phenol–chloroform method by Chomczynski and Sacci [15]. The cells (1 × 10^6^) were homogenized with 1 ml of TRI Reagent® (Merck, Poznan, Poland). The isolation protocol was performed accordingly to the protocol provided with the reagent. The quality and quantity of obtained RNA were verified spectrophotometrically using an Epoch Take3 plate (Biokom, Poznan, Polska). Prior to the reverse transcription, the RNA preparations were purified from genomic DNA by DNAse I treatment (Precision DNAse kit, Primerdesign, Blirt DNA). The cDNA synthesis for mRNA level measurement was performed using Tetro cDNA Synthesis Kit (Bioline Reagents Limited, London, UK), while for miRNA with the Mir-X ™ miRNA First-Strand Synthesis Kit (Takara Clontech Laboratories, Biokom, Poznań, Poland). The purification and reverse transcription were performed in T100 Thermal Cycler (Bio-Rad, Hercules, CA, USA). Transcripts accumulation was monitored in real-time.

The reaction was performed using SensiFast SYBR & Fluorescein Kit (Bioline Reagents Ltd.) on CFX Connect Real‐Time PCR Detection System (Bio‐Rad, Hercules, CA, USA). The total volume of the reaction mixture was 10 µl, the concentration of primers in each reaction was 400 nM, while cDNA did not exceed 1% of the mix. The Cq values obtained for genes/miRNA of interest were normalized to the reference transcripts using the previously described RQ_MAX_ algorithm. The list of used primers with the sequences and their characteristics were presented in Table S1 (Supporting Information).

### Immunodetection of intracellular biomarkers associated with histiocytosis progression by Western Blot

For the analysis sl-pHCs (1 × 10^6^) were lysed using ice-cold RIPA buffer containing 1% of protease and phosphatase inhibitor cocktail (Sigma Aldrich/Merck, Poznan, Polska). The detection was performed using the protocol established previously [8] with minor changes. Before the protein separation in 12.5% sodium dodecyl sulphate–polyacrylamide gel electrophoresis (SDS-PAGE), protein concentration was determined in samples using the Bicinchoninic Acid Assay Kit (Sigma Aldrich/Merck, Poznan, Poland). The samples were normalized for protein concentration.

The 20 μg of protein in each specimen was mixed with 4 × Laemmli loading buffer (Bio-Rad, Hercules, CA, USA) and incubated for 5 min at 95 °C in T-100 Thermal Cycler (Bio-Rad, Hercules, CA, USA). SDS-PAGE was performed at 100 V for 90 min in Mini-PROTEAN Tetra Vertical Electrophoresis Cell (Bio-Rad, Hercules, CA, USA). Separated proteins were transferred into polyvinylidene difluoride membrane (PVDF,Bio-Rad,, Hercules, CA, USA) in 1 × Transfer buffer (Tris-base/Glycine/Methanol, Sigma-Aldrich/Merck, Poznan, Poland). The transfer was performed with the Mini Trans-Blot® system (Bio-Rad, Hercules, CA, USA) at 100 V for 60 min. After the protein transfer, the membranes were blocked for 60 min at room temperature. The blocking buffer consisted of 5% bovine serum albumin (BSA) in TBS-T (Tris-buffered saline with 0.1% Tween® 20 detergent). The membranes were incubated overnight at 4 °C with primary antibodies against the protein of interest. The incubation of membranes with the secondary antibody was performed for 60 min at room temperature. The membranes were washed five times for 5 min with TBS-T buffer and gentle shaking. Table S2 (Supporting Information) lists antibodies used for the immunodetection.

### Evaluation of drugs cytotoxicity

To determine the cytotoxicity of vemurafenib (VEM) and trametinib (TRAM), cells were seeded at a density equal to 2.5 × 10^3^ on a 96-well plate in CGM. Before the assay, cells were cultured for 24 h in a CO_2_ incubator. Then, the complete growth medium (CGM) was replaced with CGM containing VEM or TRAM at a concentration equal to 100, 50, 25, 10, 5, 2.5, 1, 0.5, 0.25, and 0.1 nM. The cells were incubated with the drugs for 48 h. Then the MTS assay was performed accordingly to the manufacturer’s protocol and with the details described above. Here, the background signal was subtracted, and all values were normalized to DMSO-treated controls. Furthermore, the IC50 value, signifying the concentration at which the drug exerts half of its maximal inhibitory effect, was calculated for each cell line using Quest Graph™ IC50 Calculator (AAT Bioquest, Inc., Sunnyvale, CA, USA) [16].

### Statistical analysis

The obtained data are presented as the mean with standard deviation (± SD). Each analysis was performed with at least three technical repetitions/measurements. The comparative statistics included a one-way analysis of variance with Tukey’s post hoc test. All the calculations were performed using GraphPad Software (Prism 8.20, San Diego, CA, USA). Differences with a probability of *p* < 0.05 were considered significant.

## Results

### The karyotype and aCGH analysis

Cytogenetic analysis of GTG-banded metaphases at approximately 350-band resolution was performed on at least 30 cells per cell line. Conventional analysis revealed normal female karyotypes (46, XX) for HAN-1 and RAB-1 and normal male karyotypes (46, XY) for CHR-1.

The results of karyotyping and aCGH analysis are presented in Table 2. Normal aCGH results were obtained in cell lines HAN-1 and CHR-1, while RAB-1 showed mosaic, interstitial loss of chromosome 4 in 4q32.3q34.3 region of 13.86 Mb in size {arr[GRCh37] 4q32.3q34.3(168294580_182153223) × 1 ~ 2}. The loss encompasses 30 protein-coding genes, including OMIM morbid genes: PALLD (OMIM 608092), NEK1 (OMIM 604588), CLCN3 (OMIM 600580), HPGD (OMIM 601688), VEGFC (OMIM 601528) and AGA (OMIM 613228).

**Table 2:** Results of cell karyotyping

sl-pHC	GTG	aCGH result
HAN-1	46,XX	arr(X,1–22) × 2
CHR-1	46,XY	arr(X,Y) × 1,(1–22) × 2
RAB-1	46,XX	arr[GRCh37] 4q32.3q34.3(168294580_182153223) × 1 ~ 2

*sl-pHC* stromal-like precursors of histiocytes, *GTG* Giemsa-trypsin-Giemsa banding, *aCGH* array comparative genomic hybridization, *X* X chromosome, *Y* Y chromosome

### Cells phenotype

**Fig. 2:**
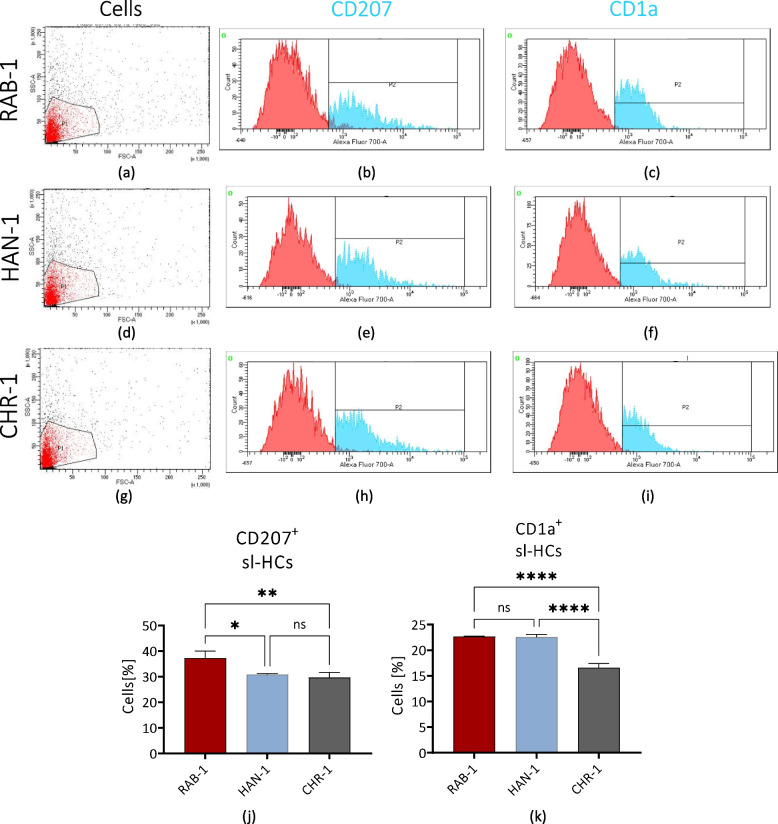
Flow cytometric analyses of sl-HCs at p3. The histiocytes markers analyzed were CD1a and CD207 (langerin). The study was performed for RAB-1 cells (**a**-**c**), HAN-1 (**d**-**f**), and CHR-1 (**g**-**i**). The obtained data were used for comparative analysis. Columns with bars represent mean value ± SD. Statistical differences were indicated: * *p*-value < 0.05, ** *p*-value < 0.01, and ****p-value < 0.0001, while the “ns” symbol refers to non-significant differences

The immunophenotype of sl-pHCs, i.e. expression of CD1a and CD207 markers, was evaluated using FACS (Fig. 2). Performed analysis revealed that the CD1a marker is expressed on the sl-pHCs surface, and about 22% of cells in RAB-1 and HAN-1 cultures are CD1a-positive cells. The percentage of CD1a + cells in the cultures of the CHR-1 line was significantly lower and equal to 16.6% ± 0.6 (Fig. 2c, f, i, k). The occurrence of CD207^+^ cells in the subsequent cultures of sl-HCs did not exceed 40%. The highest rate of CD207^+^ cells was noted in RAB-1 cell cultures (32.6% ± 2.8). Approximately 30% of cells in CHR-1 and HAN-1 cultures showed CD207 expression (Fig. 2b, e, h, j). Obtained data indicate that sl-HCs are a heterogenic population of cells with a presence of cells showing the phenotype of histiocytes-like cells (Fig. 2).

### Morphology and ultrastructure of sl-pHCs

**Fig. 3:**
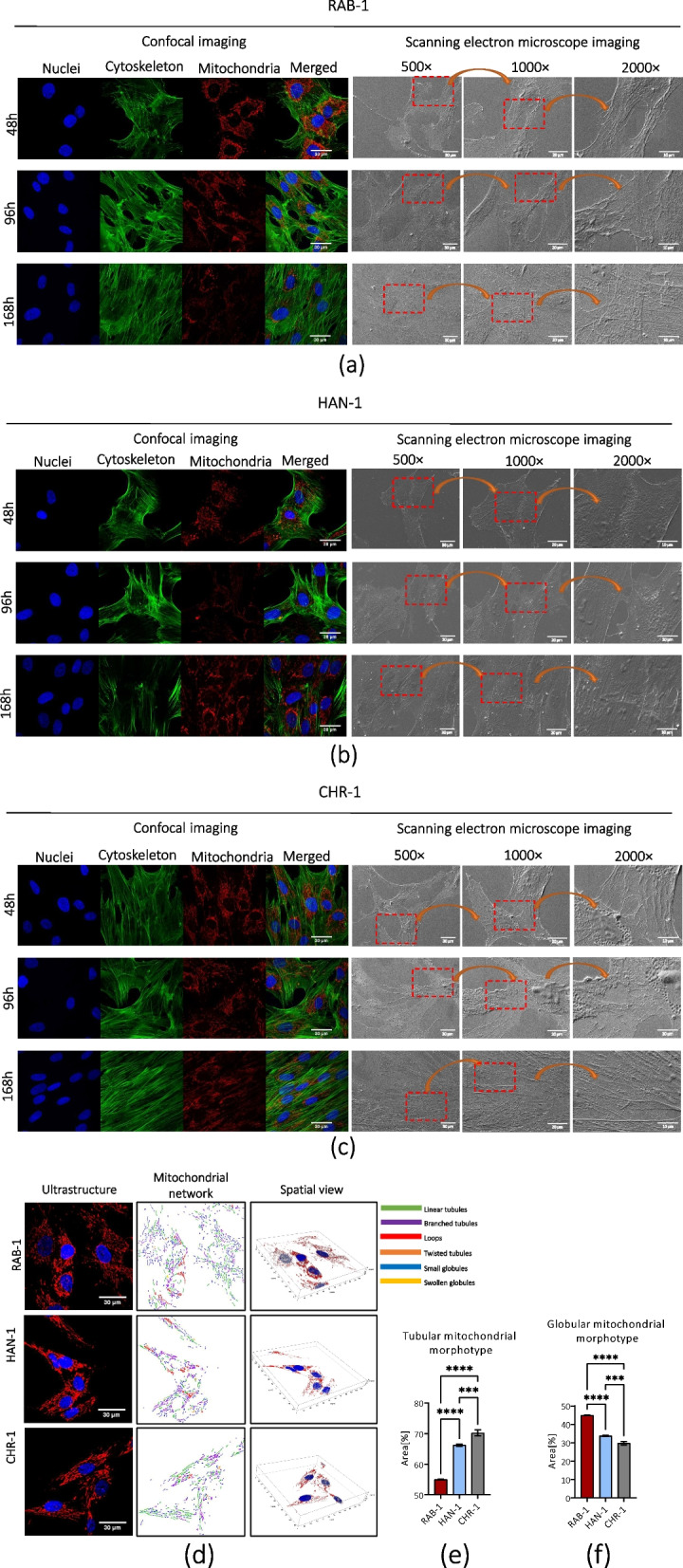
The results of the analysis aimed at evaluation of sl-pHCs’ morphology and ultrastructure. Basic cellular features, including nuclei, cytoskeleton, and mitochondrial network, were captured under a confocal microscope in sl-pHCs. In contrast, cell attachment to the surface and intercellular connections were monitored under SEM. The analysis was performed for RAB-1 (**a**), HAN-1 (**b**), and CHR-1 (**c**). Moreover, the mitochondrial network was reconstructed in 3D, and its cellular distribution and morphology were studied (**d**). The comparative analysis determined the characteristic pattern of mitochondrial morphology in the obtained sl-pHCs primary cell lines. Scale bars are indicated in the representative microphotographs. Results are presented as a column with bars representing means ± SD. *** *p*-value < 0.001, and ****p-value < 0.0001

Detailed analysis of the morphology of sl-pHCs showed that the cells are also heterogenic in shape and size (Fig. 3a-c). The stromal cell-type morphology with fibroblast-like morphotype was predominant in the subsequent cultures of sl-pHCs. All analyzed cell lines shared similar morphology and growth pattern at low density. However, RAB-1 and CHR-1, at the high confluence, formed a dense monolayer in which spindle-shaped and multipolar cells were distinguished (Fig. 3a, c). In contrast, HAN-1 in long-term culture did not create a monolayer and was characterized by multipolar, loosely interconnected cells (Fig. 3b). The confocal visualization of the actin cytoskeleton completed by SEM imaging revealed that sl-HCs have numerous outward extensions of the plasma membrane (Fig. 3a-c). These cellular projections participate in sl-pHCs adhesion to the surface and contact with adjacent cells. The overall ultrastructure characteristic of sl-pHCs cells indicated centrally located and ovoid nuclei. The mitochondrial network in all sl-pHCs cell lines was well developed, but in CHR-1 and HAN-1, tubular morphotype dominated, while the presence of globular mitochondria characterized RAB-1 (Fig. 3d-f).

### The viability and mitochondrial activity of sl-pHCs in subconfluent culture

**Fig. 4:**
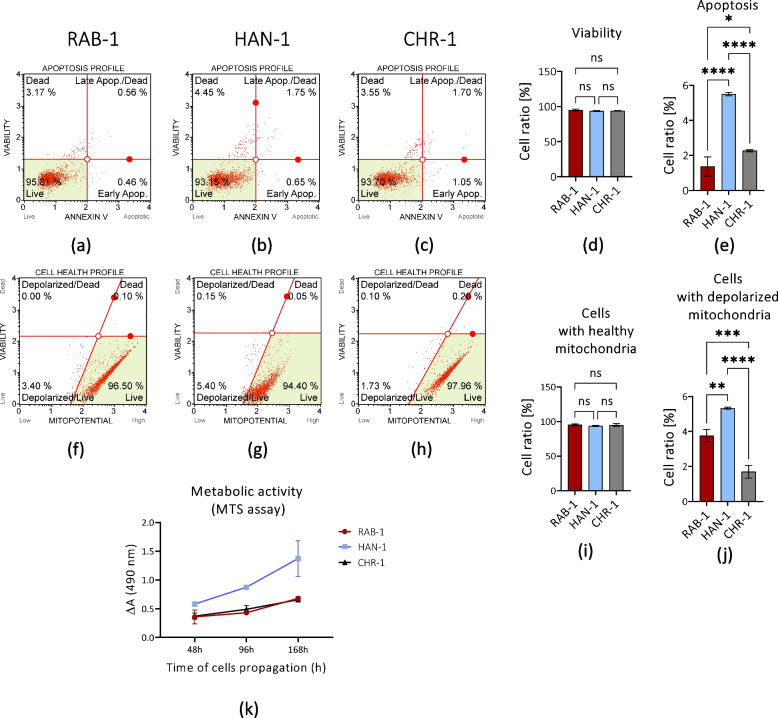
The results of assays aimed at determining sl-pHCs viability and mitochondrial activity. The cellular health of sl-pHCs was evaluated based on Annexin-V/7AAD staining to detect apoptotic cells (**a**-**e**), and mitochondrial membrane potential status (**f**-**j**). MTS assay was performed to monitor the metabolic activity of sl-pHCs in time (**k**). Columns/Boxes with bars represent mean value ± SD. Statistical differences were indicated: * *p*-value < 0.05, ** *p*-value < 0.01, *** *p*-value < 0.001, and ****p-value < 0.0001, while the “ns” symbol refers to non-significant differences

The cellular condition of sl-pHCs was determined based on the results of their staining with Annexin V/7AAD; thereby, distinguishing viable cells and apoptotic from necrotic cells was possible (Fig. 4). Analysis of sl-pHCs viability at subconfluency indicated their high cellular health (Fig. 4a-e). The percentage of viable cells reached 93%, while the apoptosis occurrence (both at an early and late stage) did not exceed 6% (Fig. 4a-e). Nevertheless, comparative analysis revealed significant differences in apoptosis incidence in sl-HCs. The most increased apoptosis occurrence was noted in the HAN-1 culture, while the lowest ratio was characteristic for RAB-1 (Fig. 4e). In general, the obtained data indicated the good cellular condition of sl-pHCs; in subconfluent culture, the cells remained healthy and viable (Fig. 4d and e).

Complementary to viability analysis, we have determined cellular health based on mitochondrial function. The high viability of sl-pHCs was linked to improved mitochondrial membrane potential, which is also in good agreement with well-developed mitochondrial network organization (Fig. 4f-j). Moreover, mitochondrial health was comparable between cell lines (Fig. 4i-j). Nevertheless, the comparative analysis showed that the HAN-1 has the highest mitochondrial injury and depolarisation occurrence, which corresponds with increased apoptosis in this cell line (Fig. 4j). At the same time, HAN-1 was characterized by the highest metabolic function reflected by succinate dehydrogenase enzymatic activity and monitored by MTS assay (Fig. 4k). In turn, the metabolism rate of RAB-1 and CHR-1 was comparable.

To conclude sl-pHCs are distinguished by increased metabolism accompanied by high mitochondrial membrane potential that corresponds with improved cellular viability (Fig. 4).

### The proliferation of sl-pHCs

**Fig. 5:**
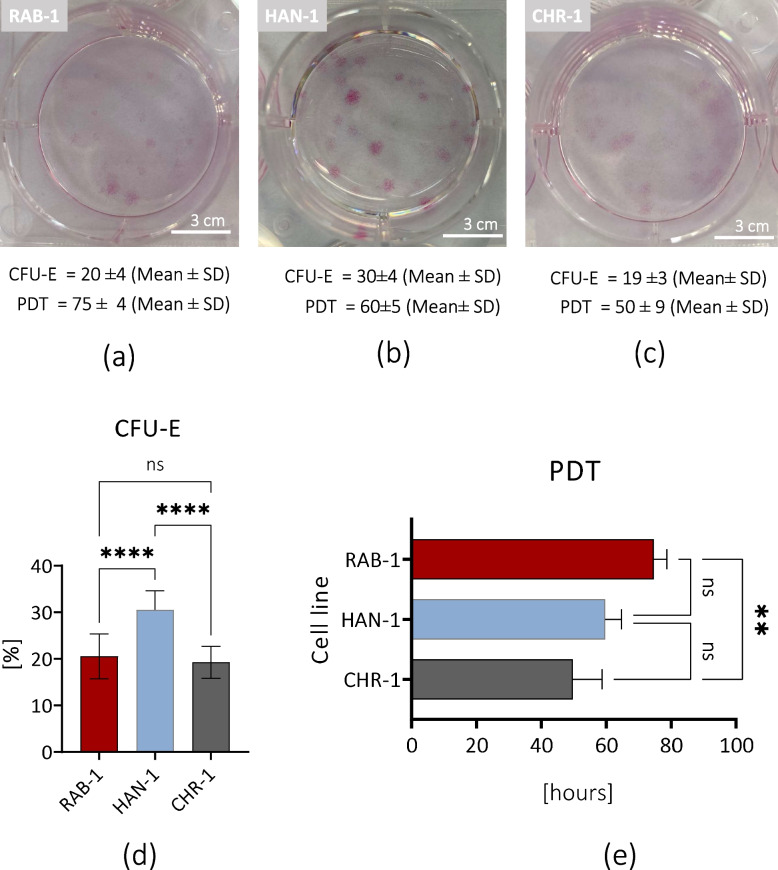
Characteristics of clonogenic activity (**a**-**d**) and population doubling time (PDT; **a**-**c**, **e**) determined for primary cultures of sl-pHCs. Results for comparative analysis are presented as columns with bars representing mean value ± SD. Statistical differences were indicated: ** *p*-value < 0.01, and ****p-value < 0.0001, while the “ns” symbol refers to non-significant differences

**Fig. 6:**
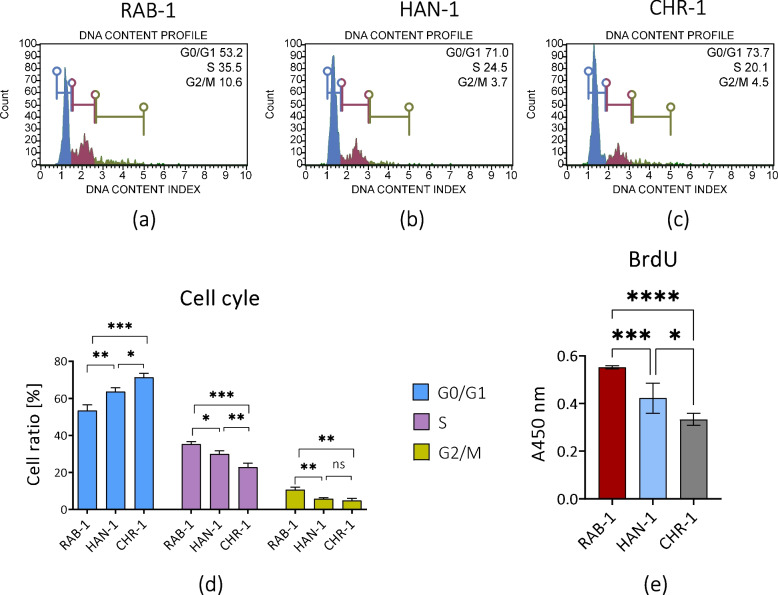
The results of tests aimed at evaluation of sl-pHCs proliferative activity. Representative histograms show the distribution of sl-pHCs in the cell cycle (**a**-**c**). Obtained data were used for comparative analysis (**d**). The proliferation of sl-pHCs was also assessed in the BrdU assay (**e**). Statistical differences were indicated: * *p*-value < 0.05, ** *p*-value < 0.01, *** *p*-value < 0.001, and ****p-value < 0.0001, while the “ns” symbol refers to non-significant differences

The proliferation of sl-pHCs was firstly analysed concerning their clonogenic activity and establishing population doubling time (Fig. 5) and complemented by analysis of cells distribution within cell cycle, as well as active DNA synthesis (Fig. 6). The analysis of the clonogenic potential of sl-pHCs primary cultures at p3 showed that the highest clonogenicity characterizes the HAN-1cells generating 30 ± 4 dense colonies (Fig. 5b and d). The clonogenic potential of RAB-1 and CHR-1 cells were comparable, equalling 20 ± 4 and 19 ± 3 colonies, respectively (Fig. 5a, c and d). However, the comparative analysis showed that HAN-1 and CHR-1 need similar time for the cells’ division of cells (Fig. 5e). The HAN-1 needed 60 ± 5 h for cell doubling, and CHR-1 needed 50 ± 9 h. In turn, the RAB-1 cell line needed 75 ± 4 h for population doubling (Fig. 5a-c and e).

At the same time, the HAN-1 and CHR-1 cell lines were characterized by the increased cell arrest associated with cells’ shift from G2/M to the G0/G1 phase and reduced number of cells in the S-phase (Fig. 6a-d). Yet, the highest proliferative activity reflected by the percentage of cells in the S-phase was in RAB-1 cultures (Fig. 6a and d). The analysis of BrdU incorporation confirmed this result (Fig. 6e). The RAB-1 cell line was shown to have the highest proliferative activity associated with active cell division, while the lowest proliferation characterized the CHR-1 cells (Fig. 6e).

### The levels of transcripts potentially associated with histiocytosis progression

**Fig. 7:**
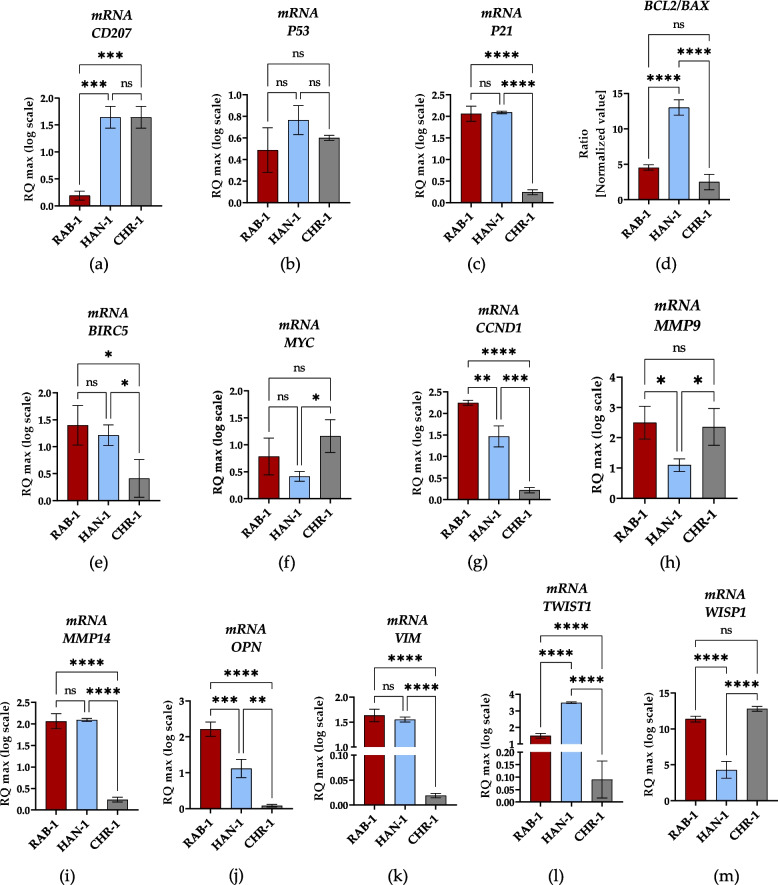
The results of RT-qPCR analysis aimed at the detection of mRNA transcripts. Results for comparative analysis are presented as columns with bars representing mean value ± SD. Statistical differences were indicated: * *p*-value < 0.05, ** *p*-value < 0.01, *** *p*-value < 0.001, and ****p-value < 0.0001, while the “ns” symbol refers to non-significant differences

**Fig. 8:**
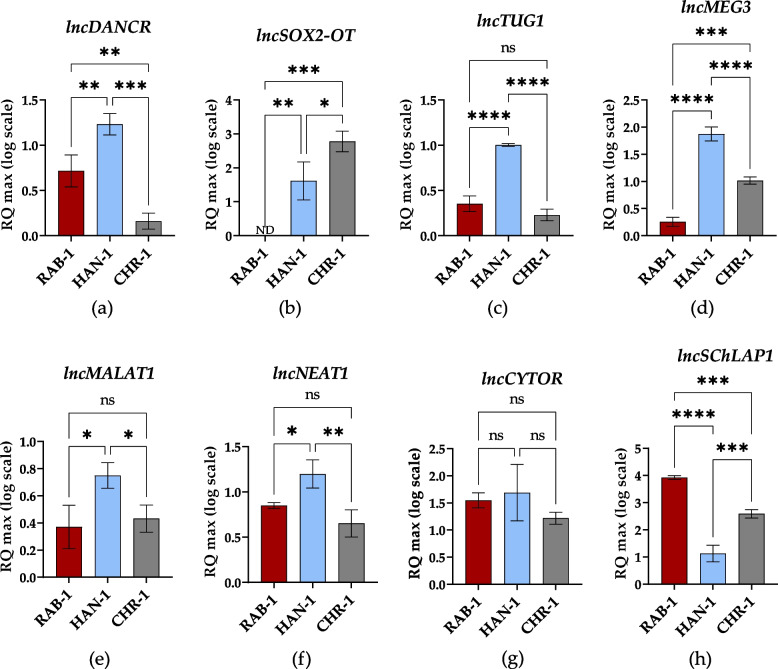
The results of RT-qPCR analysis aimed at the detection of lncRNA transcripts. Results for comparative analysis are presented as columns with bars representing mean value ± SD. Statistical differences were indicated: * *p*-value < 0.05, ** *p*-value < 0.01, *** *p*-value < 0.001, and ****p-value < 0.0001, while the “ns” symbol refers to non-significant differences

**Fig. 9:**
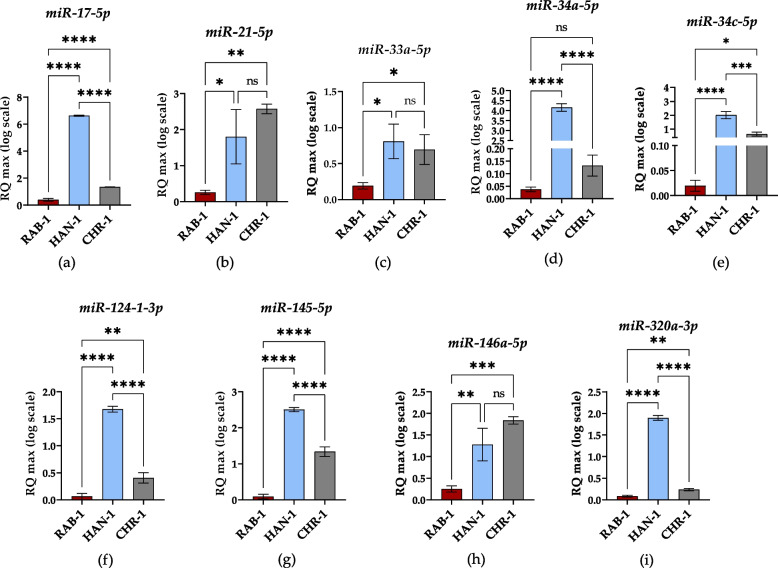
The results of RT-qPCR analysis aimed at the detection of miRNA transcripts. Results for comparative analysis are presented as columns with bars representing mean value ± SD. Statistical differences were indicated: * *p*-value < 0.05, ** *p*-value < 0.01, *** *p*-value < 0.001, and ****p-value < 0.0001, while the “ns” symbol refers to non-significant differences

The high throughput RT-qPCR assay was performed to identify the potential transcripts associated with histiocytosis progression and development. The selected molecular biomarkers were detected at mRNA (Fig. 7), lncRNA (Fig. 8), and miRNA (Fig. 9) levels.

The comparative analysis of transcript levels showed that all sl-pHCs cell lines express the mRNA for CD207. The most significant decrease in CD207 transcripts accumulation was noted in the RAB-1 cell line, while the HAN-1 and CHR-1 cells had comparable CD207 mRNA levels (Fig. 7a). Analysis of transcripts associated with cell survival indicated significant differences in mRNA expression for cyclin D (CCND1). Significantly elevated levels of CCND1 transcripts were noted in RAB-1 cells, while the lowest expression of this gene was reported in CHR-1(Fig. 7g). Moreover, RAB-1 and HAN-1 cell lines revealed common expression profiles for P21 (Fig. 7c), survivin (BIRC5, Fig. 7e), and vimentin (VIM; Fig. 7k). The mRNA levels for those transcripts were increased in RAB-1 and HAN-1 compared to CHR-1. The HAN-1 showed increased mRNA levels for anti-apoptotic BCL-2 expressed as a BCL-2/BAX-2 ratio, while RAB-1 and CHR-1 had comparable levels (Fig. 7d). No differences between cell lines were noted regarding P53 transcript levels (Fig. 7b). The CHR-1 cell line had the highest mRNA expression for MYC, but a significant difference in transcript levels was noted compared to HAN-1 (Fig. 7f).

The analysis of transcripts associated with metastasis revealed that the lowest mRNA expression for matrix metalloproteinase 9 (MMP-9) is noted in HAN-1 cells.

In contrast, transcript levels for MMP-9 in RAB-1 and CHR-1 cells were comparable(Fig. 7h). In turn, RAB-1 and HAN-1 were found to distinguish in terms of significantly increased mRNA expression of matrix metalloproteinase 14 (MMP-14) compared to CHR-1 (Fig. 7i). The expression profile of MMP-14 in the tested sl-pHCs also correlates with mRNA expression for VIM (Fig. 7i and k). Furthermore, the highest expression of OPN was noted in RAB-1 cells, while the lowest was in CHR-1 (Fig. 7j). We have also evaluated the levels of pleiotropic transcripts – TWIST-1 (Fig. 7l) and WISP-1 (Fig. 7m), both associated, i.a., with cancer cell development, viability, and metastasis. The analysis showed that HAN-1 has the highest mRNA levels for TWIST-1, while the lowest for WISP-1. Contrarily, the increased levels of WISP-1 transcript, with simultaneously the lowest levels of TWIST-1, were noted in CHR-1 cells. The WISP-1 mRNA levels were comparable between RAB-1 and CHR-1 cells (Fig. 7l and m).

The analysis of lncRNA levels showed that the expression of DANCR (Fig. 8a), SOX-2-OT(Fig. 8b), MEG3 (Fig. 8d), and SCHLAP-1 (Fig. 8h) could distinguish sl-pHCs. The expression profile of those lncRNAs varied between cell lines. For instance, SOX2-OT was not detected in RAB-1 cells, while the highest expression was noted in CHR-1(Fig. 8b). The lowest levels of MEG3 (Fig. 8d) and the highest accumulation of SCHLAP-1 (Fig. 8h) transcripts also characterized RAB-1. The highest expression of lncDANCR and MEG3 (Fig. 8d), simultaneously with the lowest expression of SCHLAP-1 (Fig. 8h), was determined in HAN-1 cells. In turn, CHR-1 cells were distinguished by the lowest levels of DANCR (Fibure 8 a). We also noted that RAB-1 and CHR-1 share similar expression patterns for lncTUG1 (Fig. 8c),

MALAT-1 (Fig. 8e), and NEAT-1 (Fig. 8f). In turn, those transcripts were significantly elevated in HAN-1 cells. No significant difference between sl-pHCs cell lines was noted regarding lncCYTOR levels (Fig. 8g).

The analysis of miRNA expression profiles indicated that sl-pHCs differ in levels for miR-17-5p (Fig. 9a), miR-34c-5p (Fig. 9e), miR-124–1-3p (Fig. 9f), miR-145-5p (Fig. 9g), and miR-320a-3p (Fig. 9i). The accumulation of those transcripts was significantly decreased in RAB-1 cells, while their highest expression was noted in HAN-1 cells. The HAN-1 and CHR-1 cell lines had comparable levels of miR-21-5p (Fig. 9b), miR-33a-5p (Fig. 9c) and miR-146-5p (Fig. 9h), while those transcripts were significantly decreased in RAB-1 cells. Moreover, the miR-34a-5p levels were downregulated in RAB-1 and CHR-1 cells while upregulated in HAN-1 cells (Fig. 9d).

Obtained data allows us to indicate transcripts distinguishing sl-pHCs and point to potentially new, functional molecules associated with histiocytosis progression.

### Intracellular accumulation of proteins associated with histiocytosis progression

**Fig. 10:**
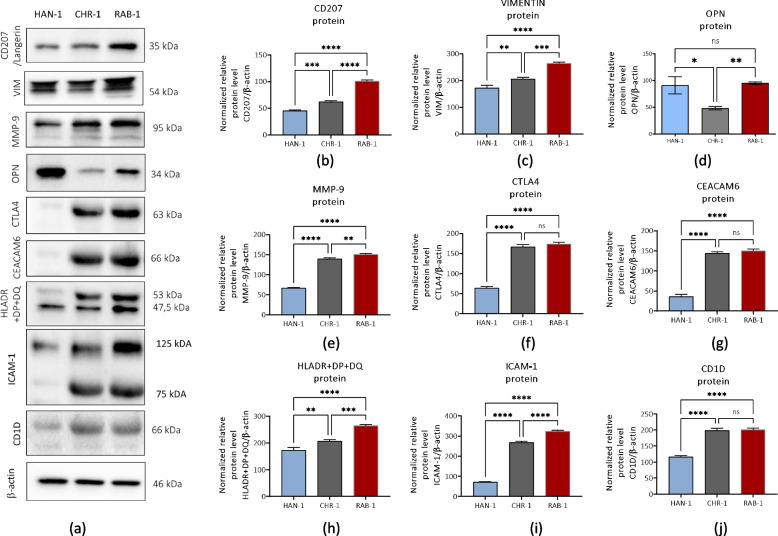
The results of immunodetection of intracellularly accumulated proteins. The representative immunoblots (**a**) show bands characteristic for detected proteins. A comparative analysis was performed to analyse the difference in the expression of histiocytosis-related biomarkers, including CD207 (**b**), vimentin (**c**), osteopontin (**d**), MMP-9 (**e**), CTLA4 (**f**), CEACAM6 (**g**), HLADR + DP + DQ (**h**), ICAM-1 (**i**), and CD1D (**j**). Results are presented as columns with bars representing mean value ± SD. Statistical differences were indicated: * *p*-value < 0.05, ** *p*-value < 0.01, *** *p*-value < 0.001, and ****p-value < 0.0001, while the “ns” symbol refers to non-significant differences

The immunodetection aimed to determine several markers associated with histiocytosis development and progression (Fig. 10). The analysis showed that the RAB-1 cell line has the highest expression of CD207 (Fig. 10a and b), vimentin (VIM; Fig. 10a and c), metalloproteinase 9 (MMP-9; Fig. 10a and e), HLADR + DP + DQ (Fig. 10a and h), and intercellular adhesion molecule 1 (ICAM-1; Fig. 10a and i). The lowest expression of those biomarkers was noted in the HAN-1 cell line. Furthermore, the RAB-1 and CHR-1 showed a significant increase in the expression of CTLA4 (Fig. 10a and f), CD1D (Fig. 10a and j) and CEACAM6 (Fig. 10a and g), over the levels noted for the HAN-1 cells.

### Drug sensitivity of sl-pHCs

**Fig. 11:**
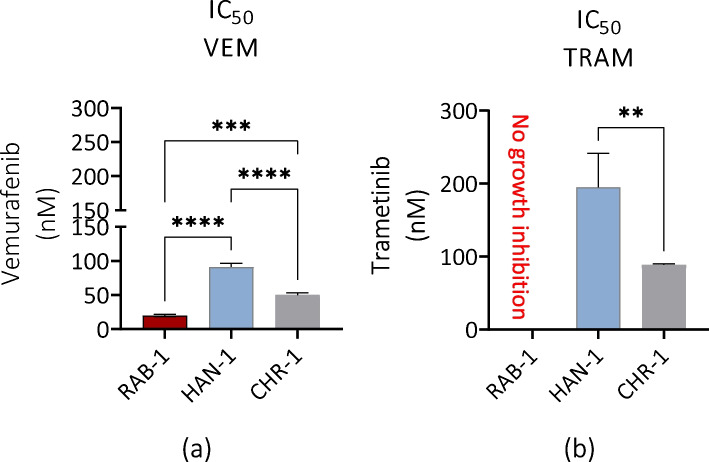
Comparison of IC50 values for vemurafenib (**a**) and trametinib (**b**) using a model of sl-pHCs. IC50 values are presented as columns with bars representing mean value ± SD. Statistical differences were indicated: ** *p*-value < 0.01, *** *p*-value < 0.001, and ****p-value < 0.0001, while the “ns” symbol refers to non-significant differences

The obtained sl-pHCs were used to determine the cytotoxicity of vemurafenib and trametinib (Fig. 11). The analysis showed that all cell lines are sensitive to vemurafenib (Fig. 11a).

The RAB-1 cell line was identified as the most susceptible to vemurafenib. Simultaneously, these cells showed no growth inhibition when treated with trametinib (Fig. 11a and b). The most sensitive cell line toward trametinib was CHR-1 (Fig. 11b). Additionally, using the model of HAN-1 and CHR-1 cells, we noticed that trametinib, on average, was two times less potent than vemurafenib (Fig. 11a and b).

## Discussion

It was previously shown that primary cell lines isolated from lesions affected by histiocytosis might play a vital role in studying the biology and pathology of the disease. The cells derived from tissues of patients with Langerhans cell histiocytosis (LCH) were described as heterogenic in terms of morphology and immunophenotype. The stromal-like cells derived from histiocytosis lesions shared fibroblast-like morphology and showed expression of marker typical for mesenchymal cells, i.e. vimentin [6, 7]. It is assumed that those cells may promote the development of granulomatous lesions and activate Langerhans cells (LCs) in situ, thus may constitute the population of progenitor cells [6, 7, 17]. Cellular models, such as DOR-1 and PRU-1 cell lines, were presented as valuable tools for testing new drugs for histiocytosis patients or models that could be used to develop personalized medicine strategies for LCH [6, 7]. Yet, primary cell lines sharing characteristics of stromal cells were mainly described based on phenotype and morphology features. While the potential strategy for treating histiocytosis could also involve targeting the stromal cells within the lesion microenvironment. Hence, employing stromal-like cells derived from histiocytic lesions as a model to develop novel therapeutic approaches for histiocytoses treatment appears evident and requires more extensive studies. Examining the efficacy of therapeutic agents utilizing those models can yield crucial insights into histiocytosis research.

In this study, we have described stromal-like cells derived from LCH and non-LCH lesions, showing low CD1a and CD207 expression. We have determined and compared the cytophysiological features of primary cell lines obtained from LCH skin and bone (RAB-1 and CHR-1 cell lines) and non-LCH skin (HAN-1 cell line). As far as we know, this is the first study providing comprehensive characteristics of the proliferative activity of primary cultures derived from histiocytic lesions, including a comparison of their molecular phenotype. The novel aspect of the study also refers to the description of the mitochondrial metabolism of sl-pHCs – the analysis aimed at determining mitochondria morphology and mitochondrial membrane polarization status.

CD1a and CD207 (langerin) antigens are accepted biomarkers of Langerhans cells (LCs), which abnormally accumulate in inflammatory lesions typical for LCH [1, 18]. The origin of CD1a/CD207 + LCs and their differentiation pathways are still elusive and not fully explained. Nonetheless, it was found that somatic mutations in MAPK pathway genes are typical of pathogenic LCs accumulating in LCH lesions [18, 19]. These mutually exclusive mutations have been identified in approximately 85% of LCH cases. Among these mutations, BRAFV600E is the most common one [19].

Our study identified stromal-like cells derived from histiocytic lesions of different clinical types. The characterized cells show low expression of both CD1a and CD207 markers. The population of CD207^+^ cells in primary cultures of sl-pHCs does not exceed 40%. The highest expression of CD207 was noted in cells derived from a patient with BRAF mutation, active LCH and abnormal karyotype, i.e. RAB-1 cell line. It was previously shown that LCH show karyotypic instability what correlates with higher clonal expansion [20, 21]. Those mutations are infrequent and mostly occurring in patients with malignant histiocytosis. Nevertheless, the exact trigger behind this chromosomal instability remains an unresolved question.

The CHR-1 and HAN-1 cells had normal karyotype and also showed expression of CD207, meaning that this marker occurs on cells derived from patient's tissue without BRAF mutation and with non-LCH diagnosis (HAN-1). Additionally, we noted that the presence of CD1a-positive cells in primary cultures of sl-pHCs was not correlated with BRAF mutation, histiocytosis type, or disease outcome.

Although CD207 was considered a marker exclusively expressed in LCH, it was shown that various cytokines, such as GM-CSF, TNF, TGF-β, and BMP-7, can induce langerin expression in human progenitor cells and monocytes [22–25]. These findings suggest that CD207 could potentially be expressed by other types of dendritic cells (DCs) beyond the LC compartment. Furthermore, Bigley et al. [25] showed that CD207 expression is not limited to LCs, as at a low level, it can be expressed by a subset of nonlymphoid and lymphoid CD1c^+^ DCs. Similarly, the expression of CD1a, which is considered a principal factor of LCH, can be noted not only in LCs but also in monocytes in response to phospholipids (phosphatidylcholine) or lectins (phytohemagglutinin) and may be regulated by various factors, including temperature [26]. The presence of CD1a in various cell types, beyond just LC and LCH, suggests that its expression can be a part of broader immunological processes, and its detection should be interpreted cautiously, particularly in differential diagnosis scenarios [27, 28].

Deep profiling of LCH cell heterogeneity using single cell RNA-seq technology also indicated on significant impact of microenvironmental factors on the phenotype and gene expression profile of LCH cells [29, 30]. The LCH heterogeneity may arise from developmental processes such as cellular differentiation, de-differentiation, or transdifferentiation [29]. The fact that that LCH exhibits key characteristics common to both cancers and immune disorders, positioning it as a uniquely valuable model for biomedical studies.

However, the in vitro models resembling immunophenotype of cells arising from histocytosis lesions are limited. Previously characterised DOR-1 and PRU-1 cells were negative for CD1a and CD207 but showed the expression of mesenchymal stromal cells marker, i.e. vimentin [6, 7]. The cells described in our study also showed intracellular expression of vimentin, which is not only a specific type III intermediate filament protein typical for mesenchymal origin but also exists as a marker of highly metastatic cancers and tumour-associated stroma [30]. Vimentin expression has been observed in tissues of patients with various histiocytic disorders, including LCH, histiocytic sarcoma (HS), interdigitating dendritic cell sarcoma (IDCS) [31, 32] and multicentric reticulohistiocytosis (MR) [33]. The cells obtained in our study also showed intracellular expression of other proteins considered biomarkers related to the progression of histiocytoses disorders, including osteopontin (OPN), MMP-9, cytotoxic T lymphocyte antigen 4 (CTLA4), CEACAM6, HLADR + DP + DQ, ICAM-1 and CD1D.

The profound study of Allen et al. showed that CD207^+^ LCH express osteopontin, a pleiotropic cytokine produced by immune and non-immune cells and controlling myriad cellular functions, including but not limited to cell motility, adhesion, and survival. OPN affects dendritic cells in an autocrine and paracrine manner, promoting their migration and differentiation as well as shaping immune responses by regulating cytokine production. Furthermore, osteopontin secreted by DCs directly affects tumour cell proliferation, survival, and spreading, while indirectly, it facilitates tumour microenvironment formation by recruiting suppressive cells derived from the myeloid lineage [34, 35]. In addition, osteopontin produced by cancer cells in the tumour environment modulates the differentiation of resident fibroblasts and mesenchymal stem cells into cancer-associated fibroblasts [34, 36, 37]. Thus, osteopontin is recently considered an essential hallmark molecule orchestrating the dialogue between cancer and stromal cells. We have noted increased osteopontin expression at mRNA and protein levels in RAB-1 and HAN-1 cells derived from skin biopsies. Indeed, osteopontin has been found to play a role in the progression of skin tumours mediating pro-inflammatory signaling. Increased expression of OPN has been associated with enhanced invasive and metastatic capabilities of melanoma and squamous cell carcinoma cells [38].

Another marker connected with histiocytosis progression is MMP-9, also identified in CD207 cells from LCH lesions [18]. The increased expression of MMP-9 in various cancer is usually correlated with a poor disease prognosis. As an illustration, MMP-9 is regarded as a significant prognostic factor for the survival of patients with malignant fibrous histiocytoma [39]. In our experiment, the highest expression of MMP-9 protein was noted in RAB-1 cell lines derived from patients with active and multisystem LCH. However, the lowest MMP-9 expression was noted in HAN-1 cells, originating from the non-LCH patient without BRAF mutation. The findings obtained are consistent with the study by Salemi et al., wherein patients with detectable circulating-free DNA BRAFV600E mutation exhibited elevated serum levels of MMP-9 compared to individuals with undetectable BRAFV600E mutation [40].

We have also noted that CTLA4, CEACAM6, and CD1D proteins were intracellularly accumulated in cells derived from lesions, but their significant expression was noted in LCH tissues with BRAF mutation. Obtained results correspond with the study by Allen et al [18]. The CTLA4 was indicated to be an important marker of LCH. Transcriptomic analysis demonstrated that CD3^+^ T cells in the LCH lesions displayed characteristics of activated regulatory T cells (T_regs_), including the expression of CTLA4, FOXP3, and SPP1, i.e. gene coding osteopontin [18]. Furthermore, the CEACAM6 molecule, acknowledged as a prognostic indicator for survival and recurrence in various types of cancer, was also overexpressed in LCH lesions [18]. However, CEACAM6 role is not only restricted to cancer progression, but this molecule can also act as an immune checkpoint inhibitor in tumours [41]. In addition, CD1D—a cell surface glycoprotein playing a critical role in presenting lipid antigens to natural killer (NK) cells, was found to be expressed by a population of LCH 207^+^ cells noted in the lesion. The expression of CD1D in LCH lesions was also increased compared with skin-resident Langerhans cells [18]. Furthermore, it was shown that mesenchymal stem cells derived from bone marrow exert an immunomodulatory effect upregulating CD1d ^high^CD5^+^ regulatory B cells in a mouse model of autoimmune encephalomyelitis (EAE). This cellular process indicates the potential interplay between stromal cells and immunocompetent cells within the histiocytic lesion.

Furthermore, we have detected that obtained cells show intracellular accumulation of the major histocompatibility class II molecules. The HLA DR, HLA DP, and HLA DQ were hitherto acknowledged as markers of antigen-presenting cells. Moreover, its expression was upregulated during different histiocytoses, e.g. LCH [18, 42] and Erdheim-Chester disease [43]. Additionally, the mesenchymal stromal cells (MSCs) themselves are also capable of expressing HLA DR [44, 45]. Studies on this issue showed that the HLA DR expression in MSC is random and dynamic [44] and can be constitutive but also triggered by interferon-gamma (IFN-γ) [46], as well as other pro-inflammatory stimulants [45]. The data obtained again indicate the possibility of an interaction between stromal cells and histiocytes within the lesion microenvironment, especially considering that MSC proficiently may impede the development of dendritic cells and their capacity to generate pro-inflammatory cytokines and stimulate potent T-cell responses [44, 47].

As discussed previously, the morphology of stromal cells derived from histiocytoses lesions is compared to fibroblastic ones. The cells with features of histiocytes progenitors were characterized as spindle-shaped with centrally located, oval nuclei [6, 7]. Those observations are consistent with ours. Although the observed morphotypes of sl-pHCs are heterogenic, we showed that the fibroblast-like morphology dominates in cell cultures obtained from histiocytoses lesions. With the use of confocal imaging, for the first time, we could evaluate the morphology of the mitochondrial network of sl-pHCs and compare it with their cellular metabolism expressed by mitochondrial membrane potential. Mitochondrial architecture and functions are intertwined and influenced by the cellular microenvironment and nutrient availability to maintain cell viability [48, 49]. Tumour cells can modify their mitochondrial structure in reaction to particular stressors to preserve functions that support tumour phenotypes and promote malignant transformation as well as progression [50]. It also potentially impacts treatment response as the mitochondria are significant organelles linked to the resistance of chemotherapeutic drugs, and imbalances in mitochondrial dynamics are associated with variations in sensitivity to chemotherapy [48, 49]. For instance, using tamoxifen-resistant breast cell lines, Tomkova et al. found that the drug-resistant cell lines have a more fragmented mitochondrial network [51]. This observation aligns with our study, as we noted that the RAB-1 cell line, characterized by fragmented mitochondria with globular morphotype, was susceptible to vemurafenib while resistant to trametinib.

Although mounting evidence indicates that mitochondrial fission and fusion may promote cancer development, fission usually correlates with enhanced cancer cell proliferation and progression [52, 53]. Grieco et al. [50], using the MOSE cell model for serous ovarian cancer, showed that during progression, mitochondrial morphology changes from a filamentous network to single organelles with a greater degree of circularity. The smaller mitochondria noted during the progression of the MOSE cell line were also organized near the nucleus. Additionally, Grieco et al. indicated that changes in mitochondrial morphology do not appear to impact essential mitochondrial processes like apoptosis induction, respiration, or overall cell survival [50]. Notwithstanding, all sl-pHCs cell lines obtained in this study were distinguished by high cellular viability (above 93% of cells), which was also expressed by high mitochondrial membrane potential. Indeed RAB-1 cultures were characterized by the lowest occurrence of apoptotic cells but also increased proliferation defined by a high ratio of cells in the S-phase and G2/M phase of a cell cycle. According to Tanwar et al., the role of Drp1, i.e. protein that governs mitochondrial fission, may be crucial in maintaining tumorigenic cell proliferation in epithelial ovarian cancer (EOC) by mitosis boost [54]. The increased number of RAB-1 cells in the S-phase, simultaneously with the longer time needed for doubling time, can be related to the fact that cancer cell lines have longer S-phase than untransformed cells [55–57]. In turn, CHR-1 cells had lowered proliferative activity connected with increased cells in the G0/G1 phase of the cell cycle, which is also consistent with high mitochondrial membrane potential and tubular mitochondrial system characterizing that cells [58] but can also indicate their invasive potential [56].

Additionally, we have found limited research on the clonogenic activity of cells in histiocytoses. However, Chakraborty et al. determined that CD207 + cells can promote clonality of pathological DCs with high BRAFV600E/MAP2K1 variant allele fractions. While in our study, the highest clonogenic potential distinguished the HAN-1 cells, which derived from the patient without BRAFV600E mutation both in pathological tissue and blood. The clonogenic activity expresses not only the proliferative activity of single cancer cells but also is a functional assay that measures the stemness of cancer stem cells (CSCs) and the tumourigenic potential of cancer cells. The high clonogenic activity of HAN-1 cells can be related to increased expression of several long non-coding RNAs, such as TUG1, MEG3, MALAT1, NEAT1, and DANCR – all considered as modulators of stemness of cancer cells, their differentiation potential and chemoresistance [59–63].

Moreover, we have also noted that HAN-1 cells had increased mRNA expression for TWIST1, which also correlated with elevated levels of anti-apoptotic BCL-2. This profile is consistent with the results obtained by Khales et al. [64], who showed that TWIST1 might promote the stemness phenotype of esophageal squamous cell carcinoma cells suppressing their sensitivity to apoptosis via up-regulation of BCL-2 and down-regulation of BAX. The HAN-1 cells were also distinguished by elevated levels of small non-coding RNAs, namely miR-17-5p, miR-34c-5p, miR-124–1-3p, miR-145-5p and miR-320a-3p that also exists as a so-called onomiRNAs particularly involved in progression and tumorigenesis of various cancers.

In addition, the expression profile of several transcripts confirmed the high proliferative potential of RAB-1 cells. Not only mRNA levels for OPN were increased in RAB-1 cells, but also lncRNA SChLAP1. Both OPN and SChLAP1 are molecules that effectively promote the proliferation and migration of cancer cells [36, 65, 66]. Moreover, SChLAP1 improves the invasiveness of prostate cancer cells [65], and their high levels correlate with tumour aggressiveness [66].

In contrast to RAB-1 and HAN-1, the transcriptome profile of CHR-1 was distinguished by significantly lowered levels of P21, CCD1, MMP-14, VIM, TWIST1, and DANCR, which may reflect their decreased proliferation activity. In turn, significantly increased levels of lncRNA SOX2OT were noted in CHR-1 could contribute to their improved metabolism, evidenced by a developed mitochondrial network and lowered number of cells with depolarized mitochondrial membranes. In fact, Liang et al. showed that SOX2OT overexpression or knockdown in hepatocellular carcinoma did not change oxygen consumption or ATP level but may be responsible for regulating glycolysis [67].

The presented study has some limitations, firstofall only three cell lines derived from pediatric patients were analysed, which limits the generalizability of the findings. While the heterogeneity of the described cell lines (RAB-1, HAN-1, and CHR-1) offers insights into diverse manifestations of the disease, it may also complicate the interpretation of results and their application. The study may potentially miss out on other relevant cellular behaviors or molecular pathways due to the analysied cells were not compared with normal cells (macrophages and dendritic cells subtypes) and fresh tissue histiocytosis samples. Given the above, translation from cellular models to clinical application requires further research and validation in clinical settings, but at the same time the study highlights the impact and relevance of research in the field of rare diseases and cellular biology.

## Conclusions

Understanding the biology of cells derived from histiocytoses lesions is essential in testing and developing new methods of histiocytoses treatment. Since research on in vitro models of histiocytoses is limited, obtained data are original and provide novel insights into the cytophysiology of cells derived from histiocytosis-affected tissues, such as the potential significance of mitochondrial distribution and network formation in relation to drug sensitivity. Ultimately, we showed that cells derived from histiocytoses lesions share features of stromal cells maintaining low expression of CD207 and CD1a. The cell lines described in this study differ regarding proliferative activity and expression profile of biomarkers associated with histiocytoses progression.

In addition, we have identified several transcripts previously unassociated with histiocytoses. Those biomarkers can have diagnostic and prognostic value, but further functional studies will be essential to determine whether they can also serve as potential therapeutic targets in histiocytosis. Special consideration should be given to non-coding RNAs as their levels may reflect cells' proliferative and metabolic status within the histiocytic lesion. Such specific expression patterns of non-coding may also be a hallmark of different histiocytoses subtypes and thus help better identify the disease.

To summarize, we believe that the obtained and characterized models of stromal-like cells derived from histiocytic lesions can be used for in vitro studies on histiocytosis biology and drug testing.

### Supplementary Information

**Additional file 1: Table S1.** The list of used primers with the sequences and their characteristics. **Table S2.** The lists antibodies used for the immunodetection.

**Additional file 2. **

## Data Availability

The data that support the findings of this study are available from the corresponding author upon reasonable request.

## Abbreviations

AWD: Alive with disease
BAX: Bcl-2-associated X protein
BCL-2: B-cell lymphoma 2
BIRC5: Survivin
BMP-7: Bone morphogenetic protein 7
BRAFV600E: V-raf murine sarcoma viral oncogene homolog B1 V600E
BrdU: Bromodeoxyuridine
CEACAM6: Carcinoembryonic antigen-related cell adhesion molecule 6
CHT: Chemotherapy
CGM: Complete growth medium
CD1a: Cluster of differentiation 1a
CD1D: Cluster of differentiation 1d
CD207: Cluster of differentiation 207
CTLA4: Cytotoxic T-lymphocyte-associated protein 4
CYTOR: Cytoskeleton Regulator RNA
DANCR: Differentiation antagonizing non-protein coding RNA
DCs: Dendritic cells
DDC: Stromal dermal dendritic cell
EOC: Epithelial ovarian cancer
EAE: Experimental autoimmune encephalomyelitis
F: Female
GM-CSF: Granulocyte–macrophage colony-stimulating factor
HBSS: Hanks' Balanced Salt Solution
HLADR + DP + DQ: Human leukocyte antigen-DR + DP + DQ
HS: Histiocytic sarcoma
ICAM-1: Intercellular adhesion molecule-1
IDCS: Interdigitating dendritic cell sarcoma
LCs: Langerhans cells
LCH: Langerhans cell histiocytosis
LG DMEM: Low glucose Dulbecco’s Modified Eagle Medium
MAPK: Mitogen-activated protein kinase
M: Male
MALAT-1: Metastasis-associated lung adenocarcinoma transcript 1
MEG3: Maternally expressed gene 3
MMP-9: Matrix metalloproteinase 9
MMP-14: Matrix metalloproteinase-14
MR: Multicentric reticulohistiocytosis
NEAT-1: Nuclear-enriched abundant transcript 1
OPN: Osteopontin
P/S: Penicillin/streptomycin mix
P21: Cyclin-dependent kinase inhibitor 1
P53: Tumor protein p53
PDT: Population doubling time
SEM: Scanning electron microscope
sl-pHCs: Stromal-like precursors of histiocytes
SOX-2-OT: SOX2 overlapping transcript
SCHLAP-1: SWI/SNF Complex Antagonist Associated With Prostate Cancer 1
TNF: Tumor necrosis factor
TRAM: Trametinib
TGF-β: Transforming growth factor beta
TWIST-1: Twist-related protein 1
TUG1: Taurine upregulated gene 1
VEM: Vemurafenib
VIM: Vimentin
WISP-1: WNT1 inducible signalling pathway protein 1
