# Supplementary material for: Identification and characterization of stromal-like cells with CD207+/low CD1a+/low phenotype derived from histiocytic lesions – a perspective in vitro model for drug testing
Source: BMC Cancer. 2024 Feb 12;24:105. doi: 10.1186/s12885-023-11807-0 (PMC10860276; doi:10.1186/s12885-023-11807-0)
Supplement: Supplementary file 2 — Additional file 2. [file 12885_2023_11807_MOESM2_ESM.pdf]

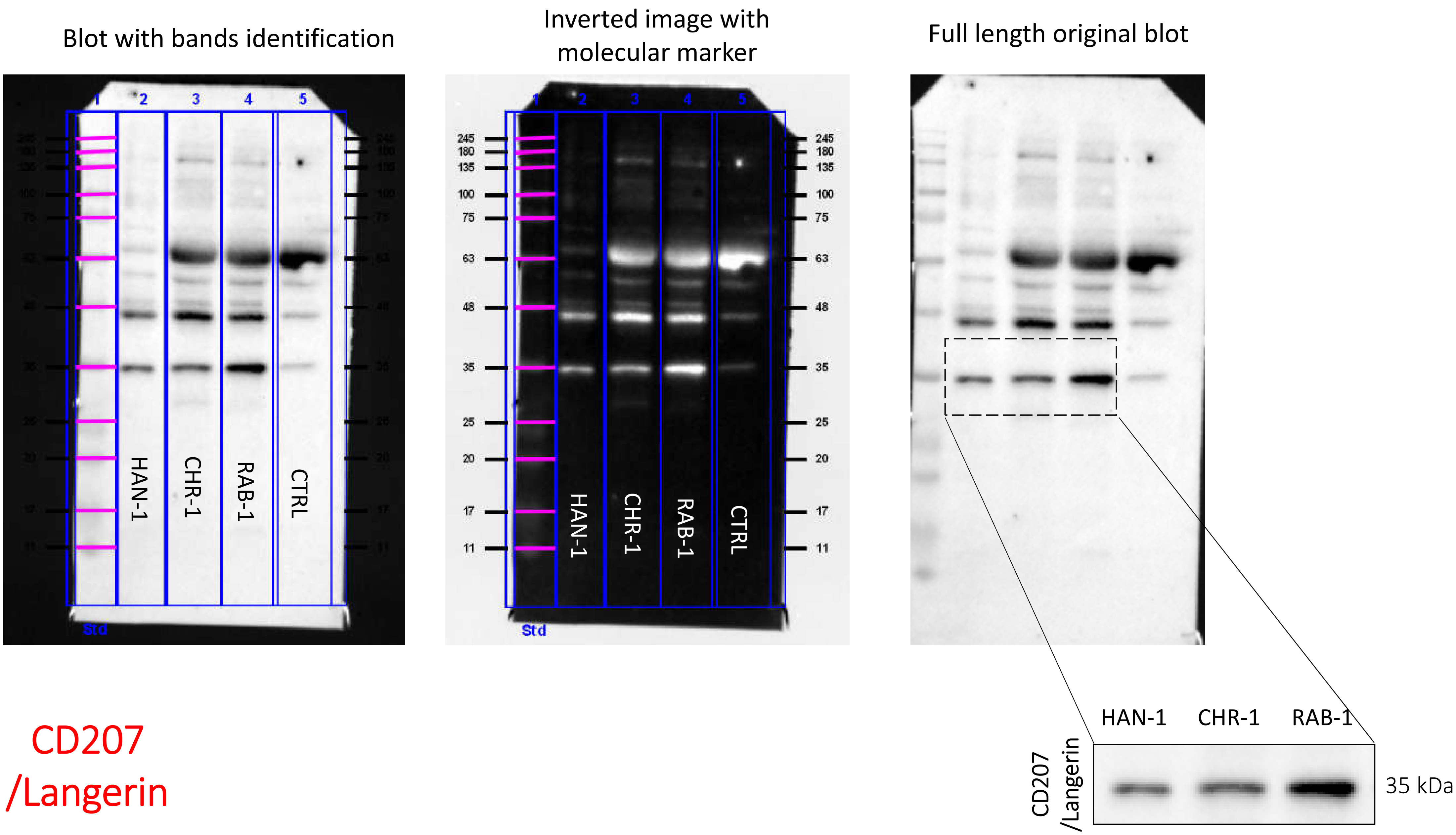

Blot with bands identification
Inverted image with molecular marker
Full length original blot
1 2 3 4 5
245
180
135
100
75
63
48
35
25
20
17
11
HAN-1
CHR-1
RAB-1
CTRL
Std
HAN-1
CHR-1
RAB-1
CD207 /Langerin
35 kDa
CD207 /Langerin

Blot with bands identification

Inverted image with molecular marker

Full length original blot
Inverted to maintain the sample location presented in publication

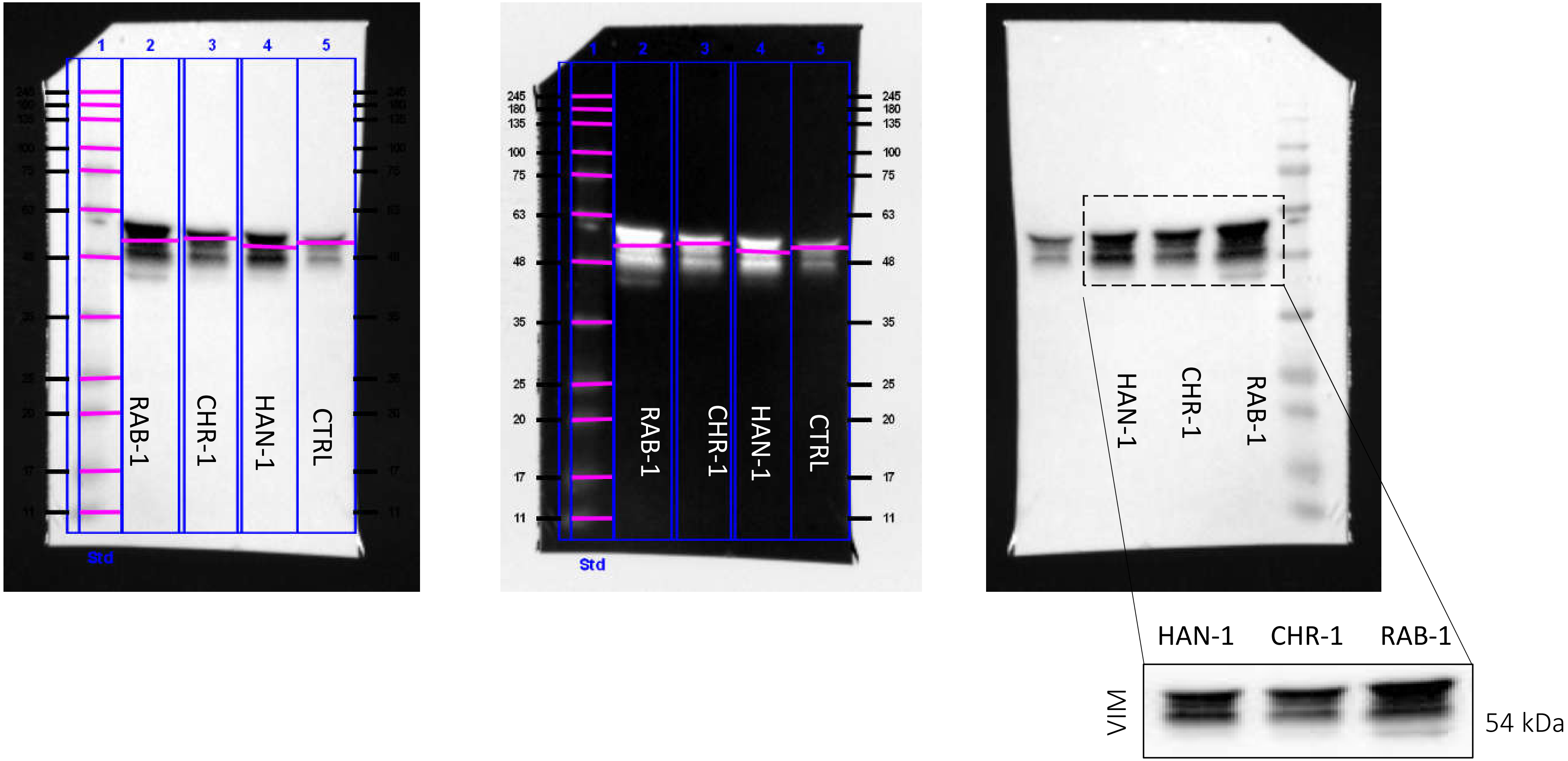

VIMENTIN

Blot with bands identification

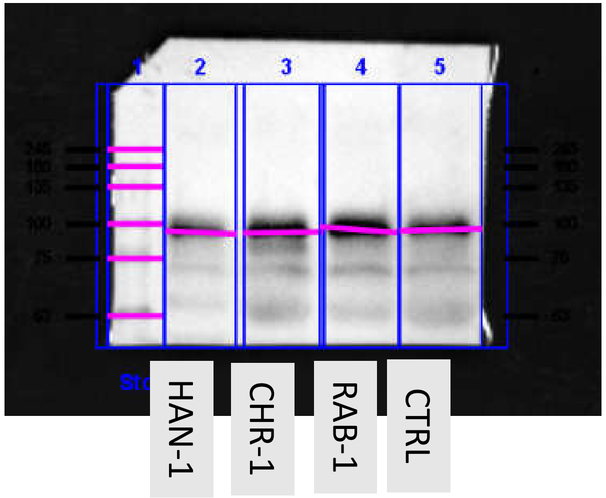

Inverted image with molecular marker

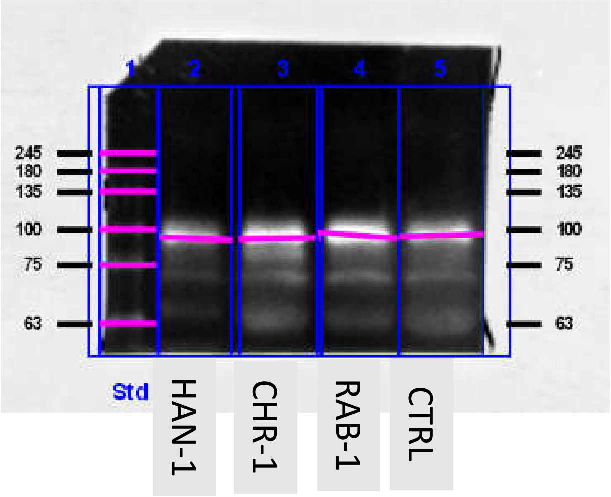

Full length original blot
Inverted to maintain the sample location presented in publication

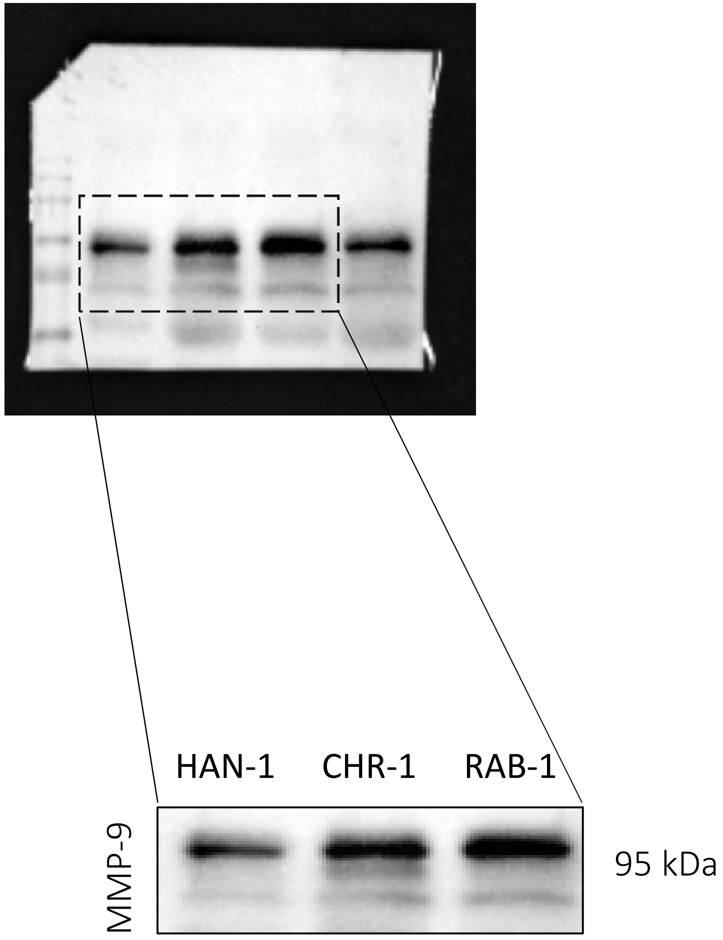

MMP-9

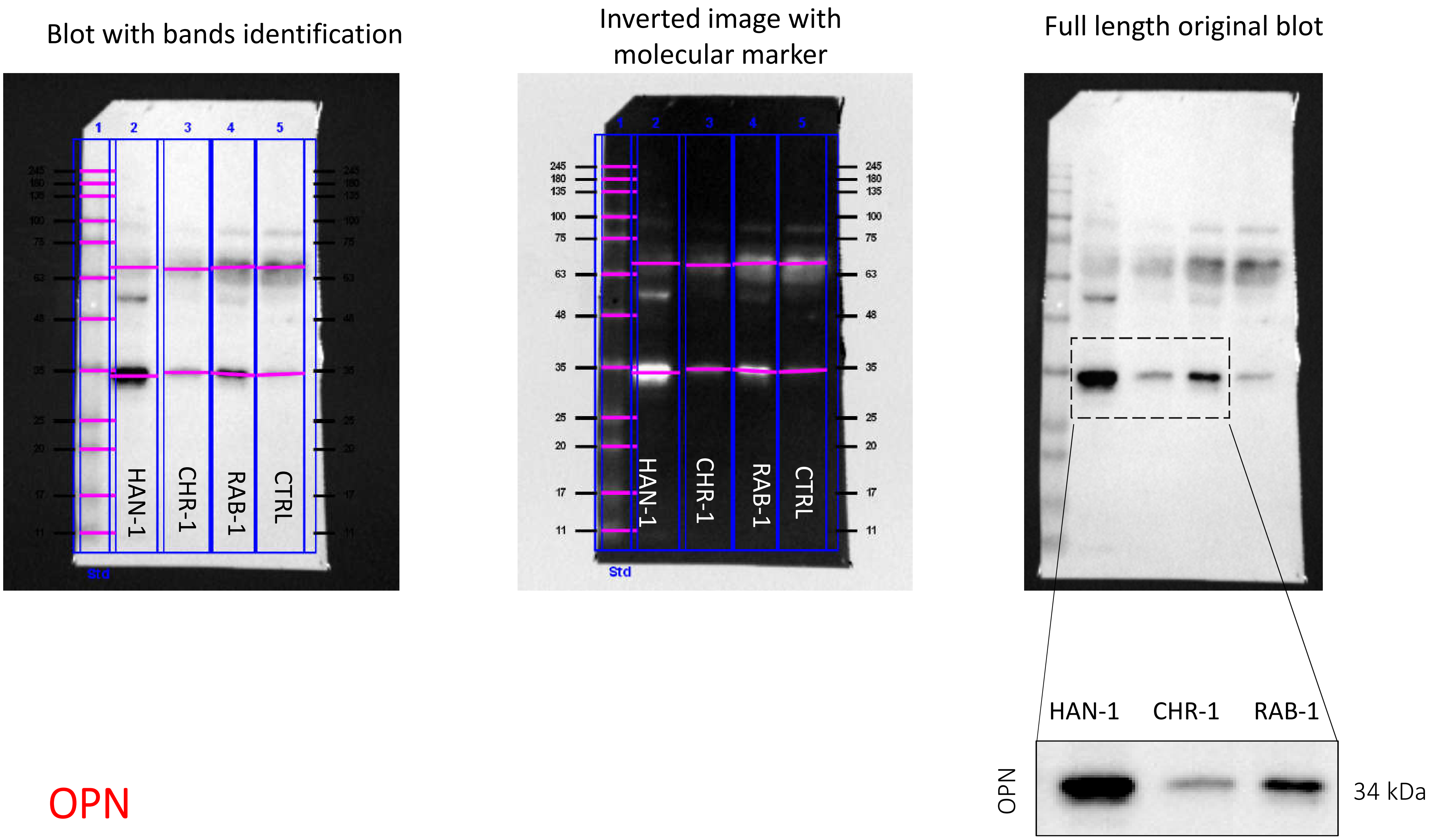

Blot with bands identification
Inverted image with molecular marker
Full length original blot
1
2
3
4
5
245
180
135
100
75
63
48
35
25
20
17
11
Std
HAN-1
CHR-1
RAB-1
CTRL
HAN-1
CHR-1
RAB-1
OPN
34 kDa
OPN

Blot with bands identification

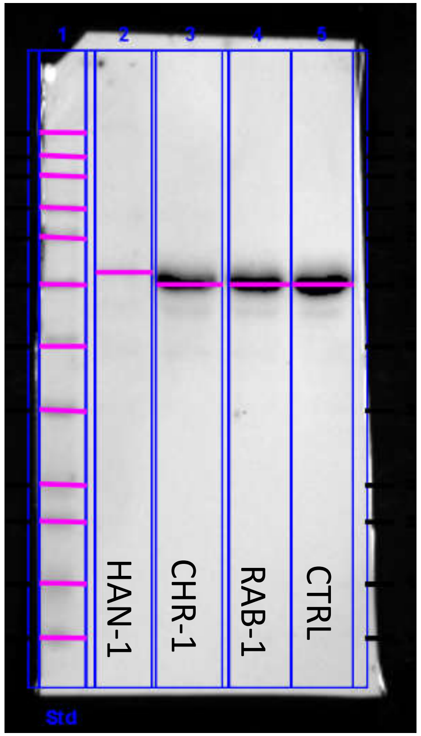

Inverted image with molecular marker

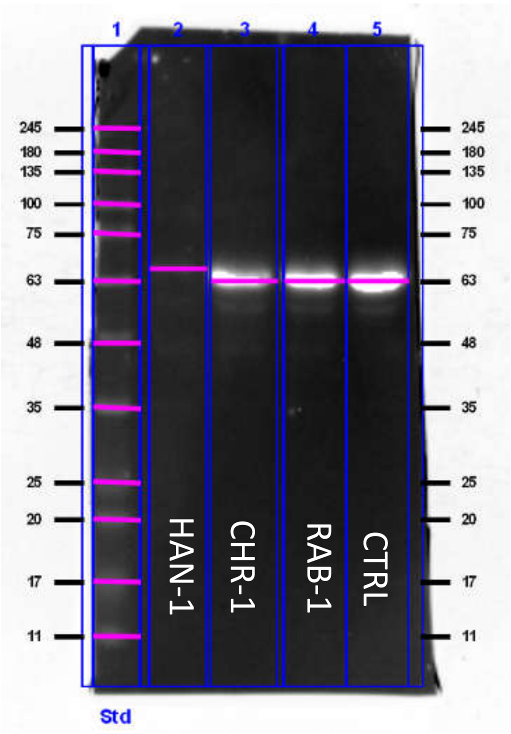

Full length original blot

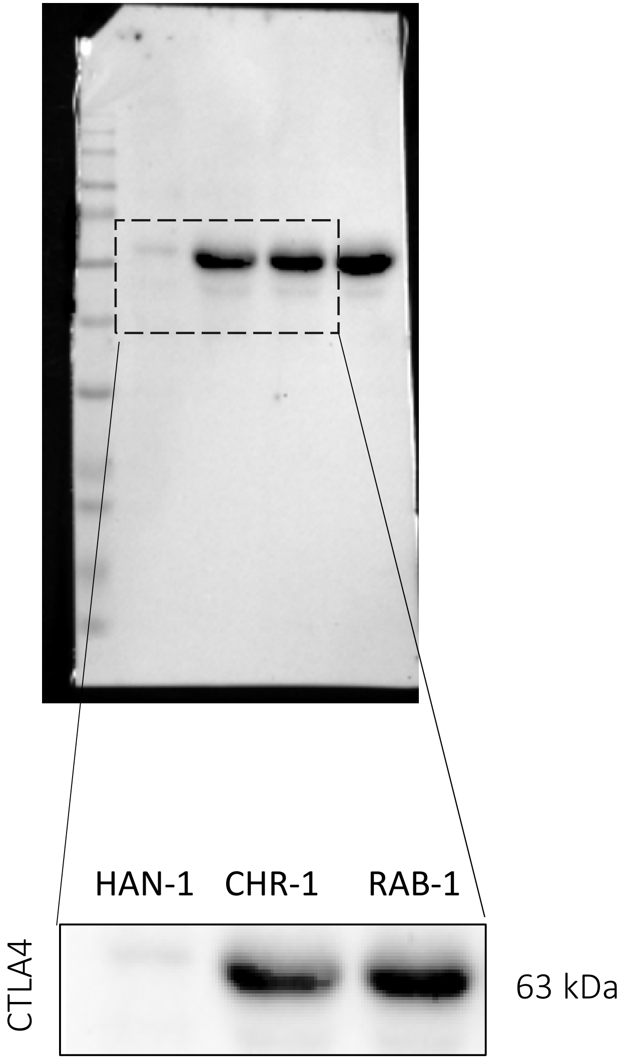

CTLA4

Blot with bands identification

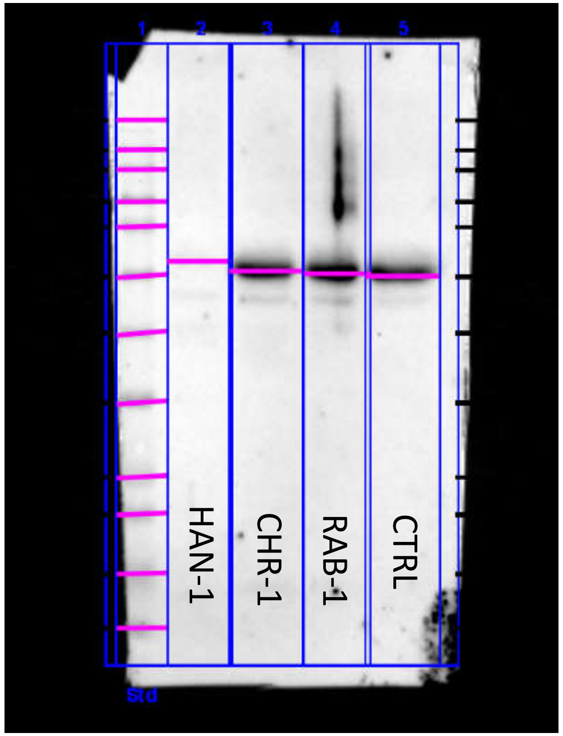

Inverted image with molecular marker

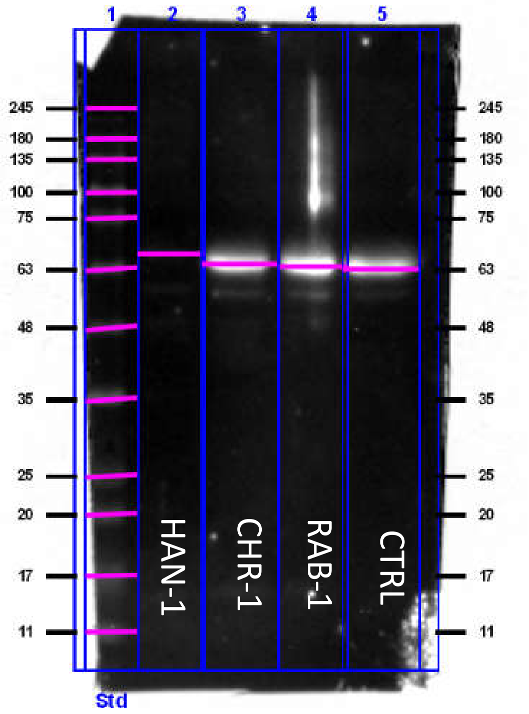

Full length original blot

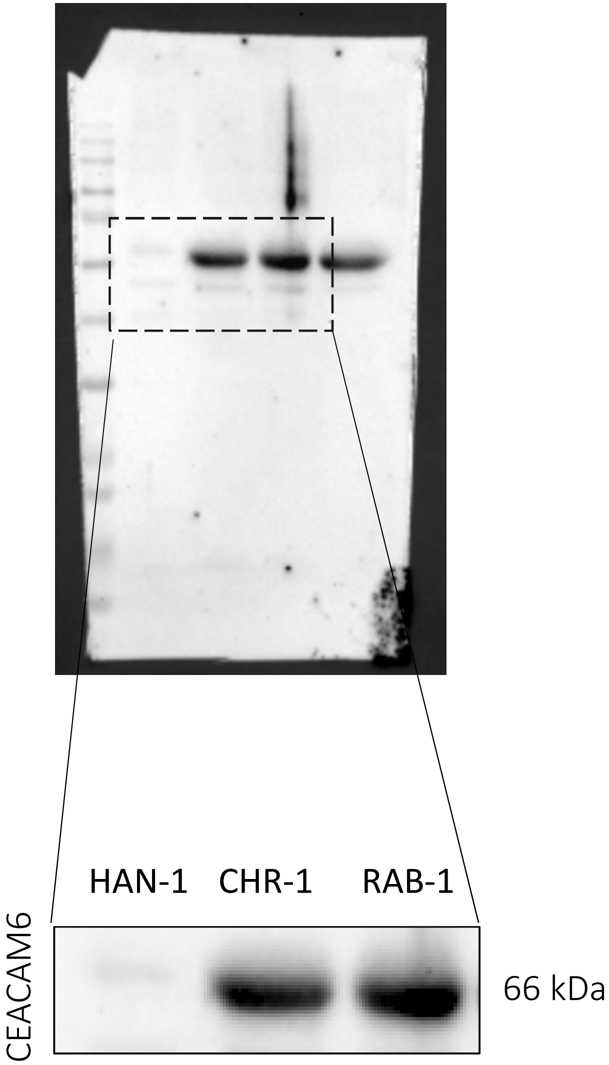

CEACAM6

Blot with bands identification

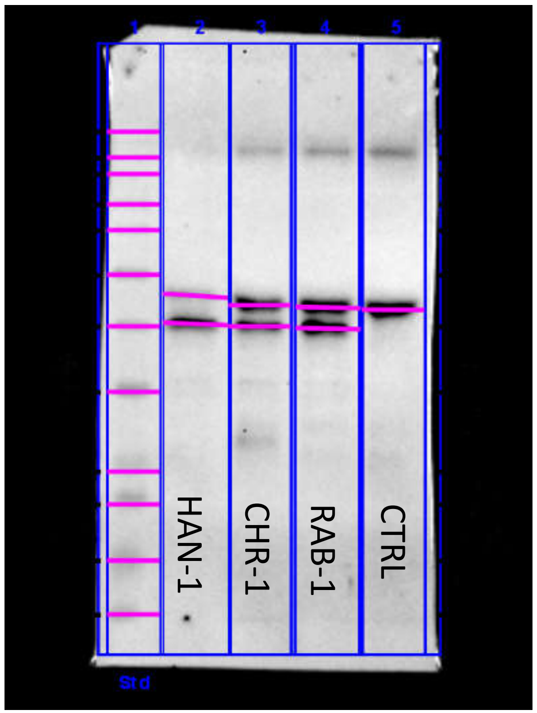

Inverted image with molecular marker

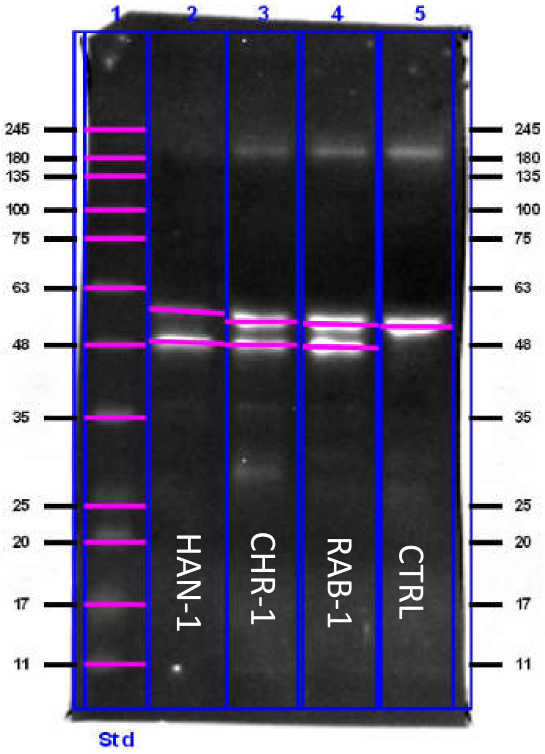

Full length original blot

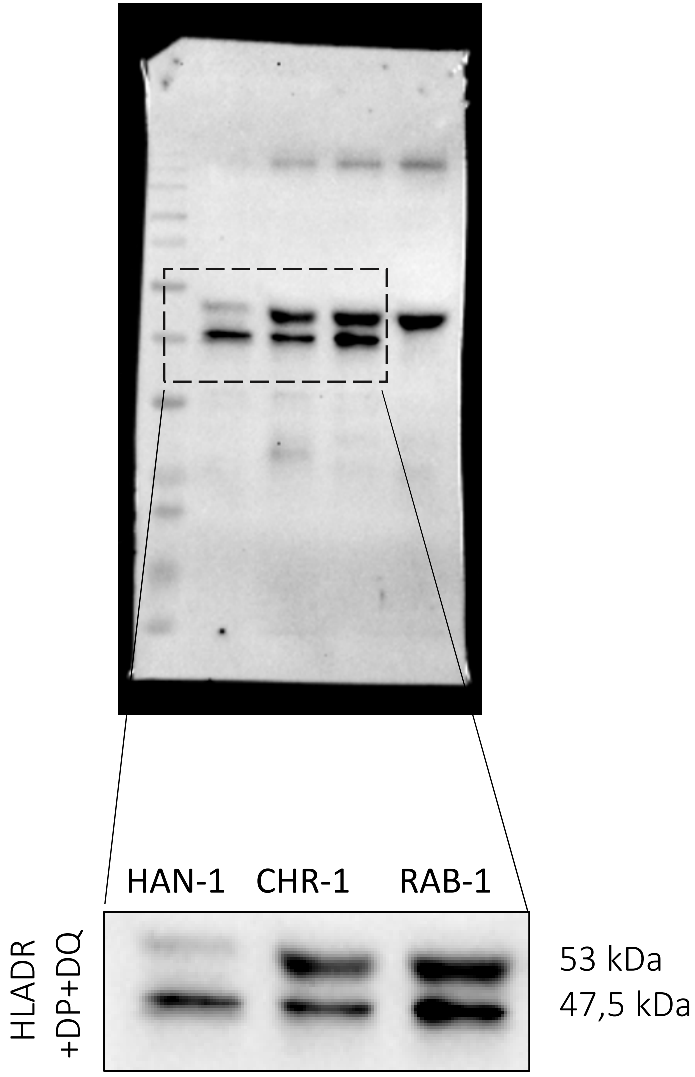

HLADR
+DP+DQ

Blot with bands identification

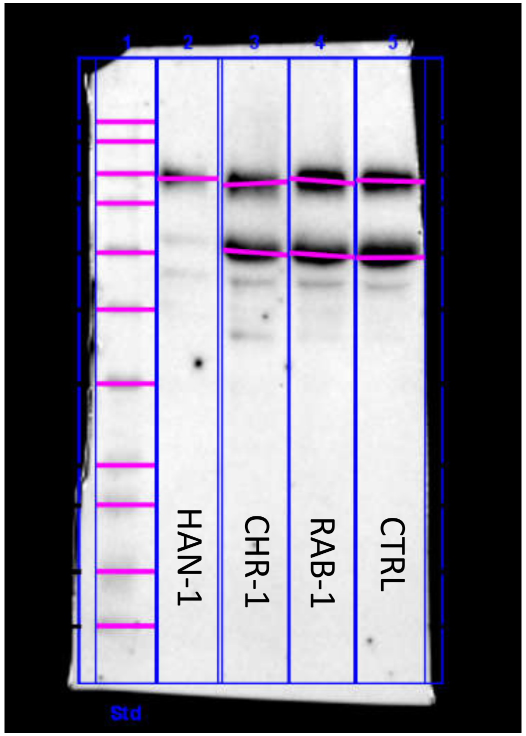

Inverted image with molecular marker

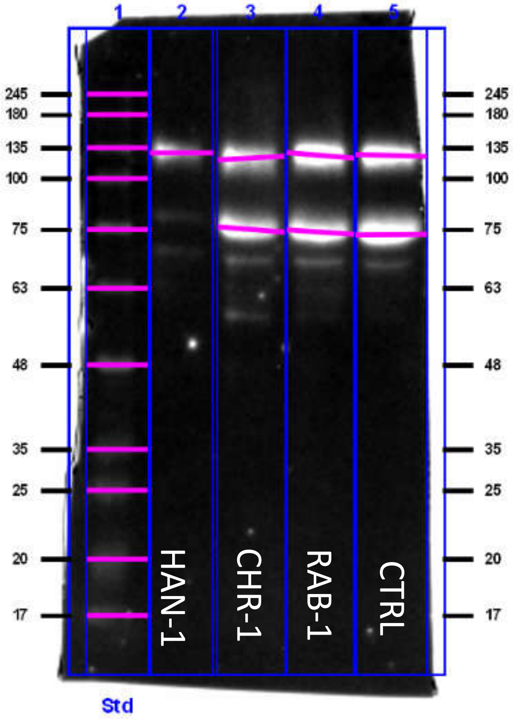

Full length original blot

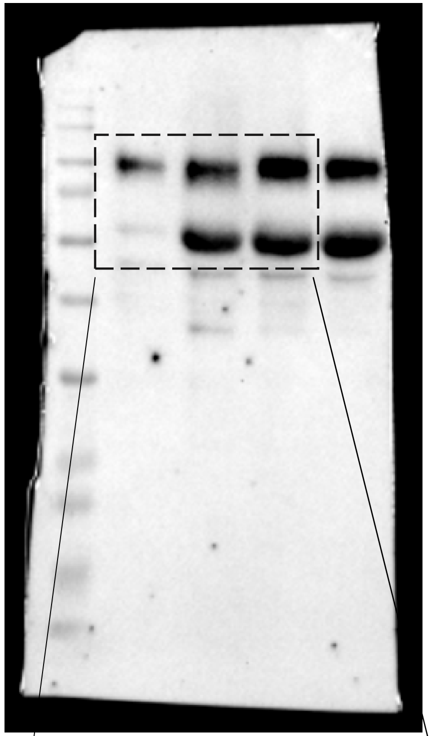

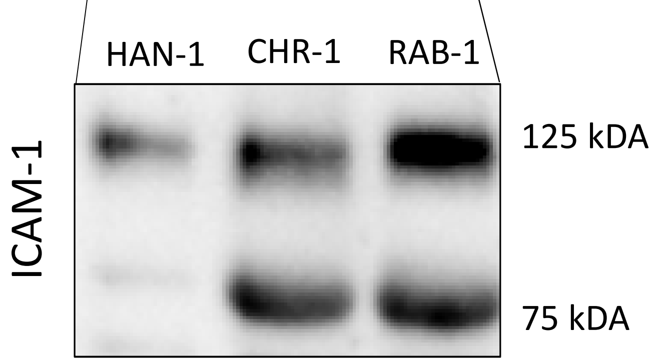

ICAM-1

Blot with bands identification

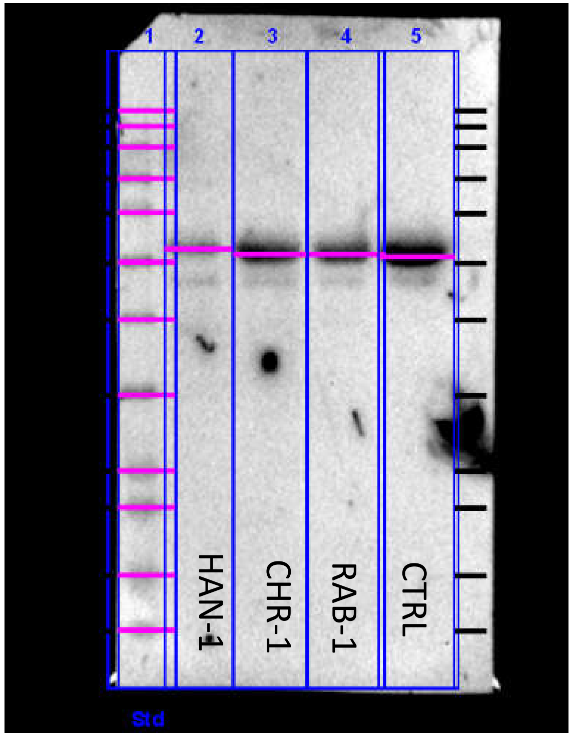

Inverted image with molecular marker

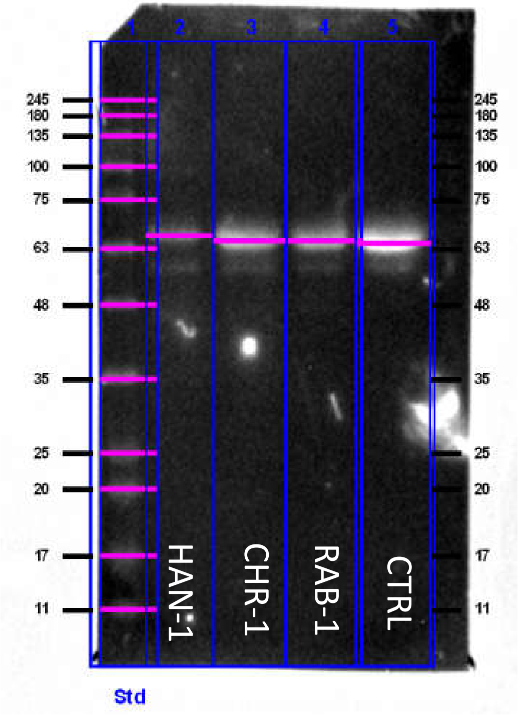

Full length original blot

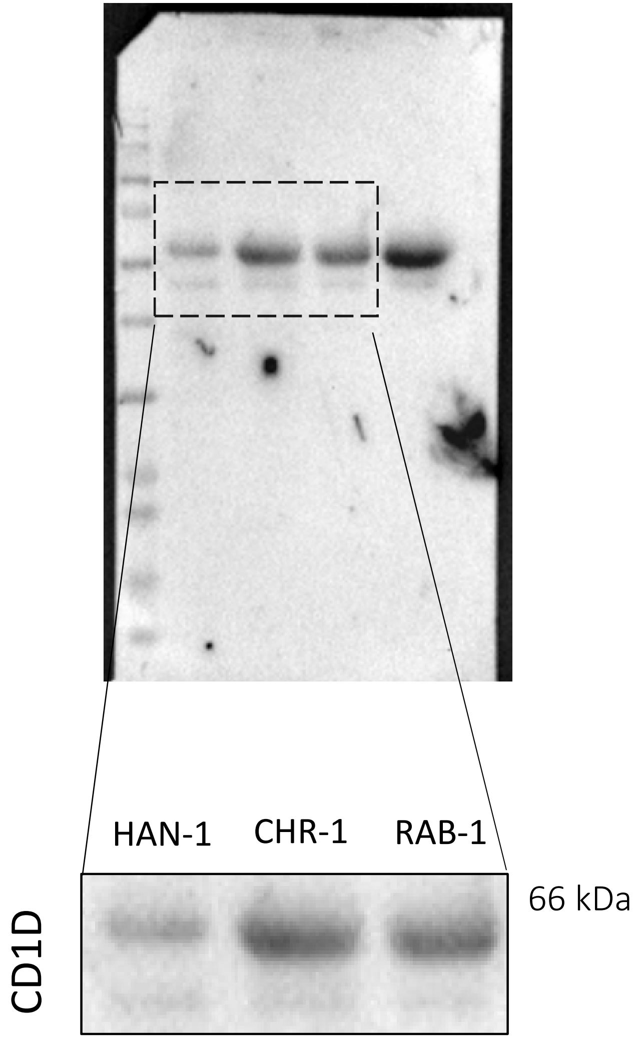

CD1D

Blot with bands identification

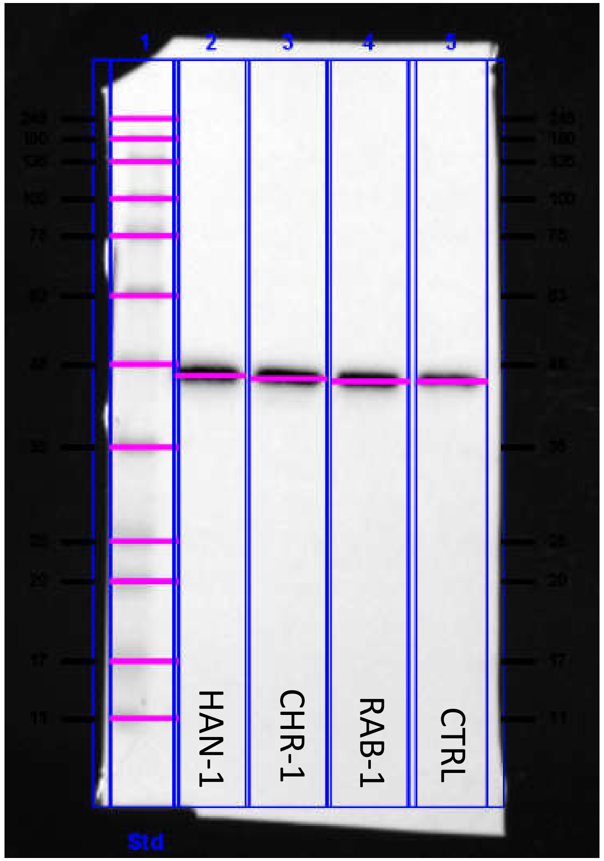

Inverted image with molecular marker

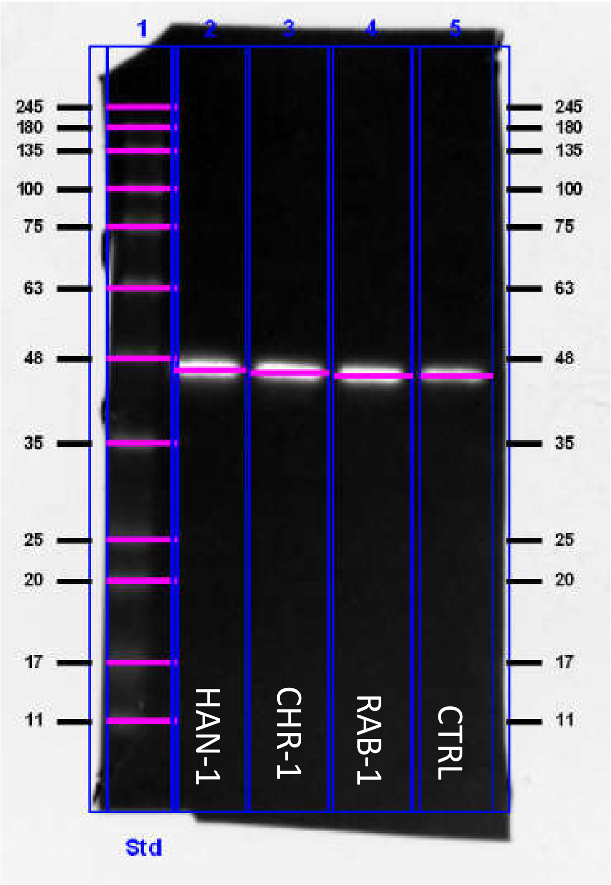

Full length original blot

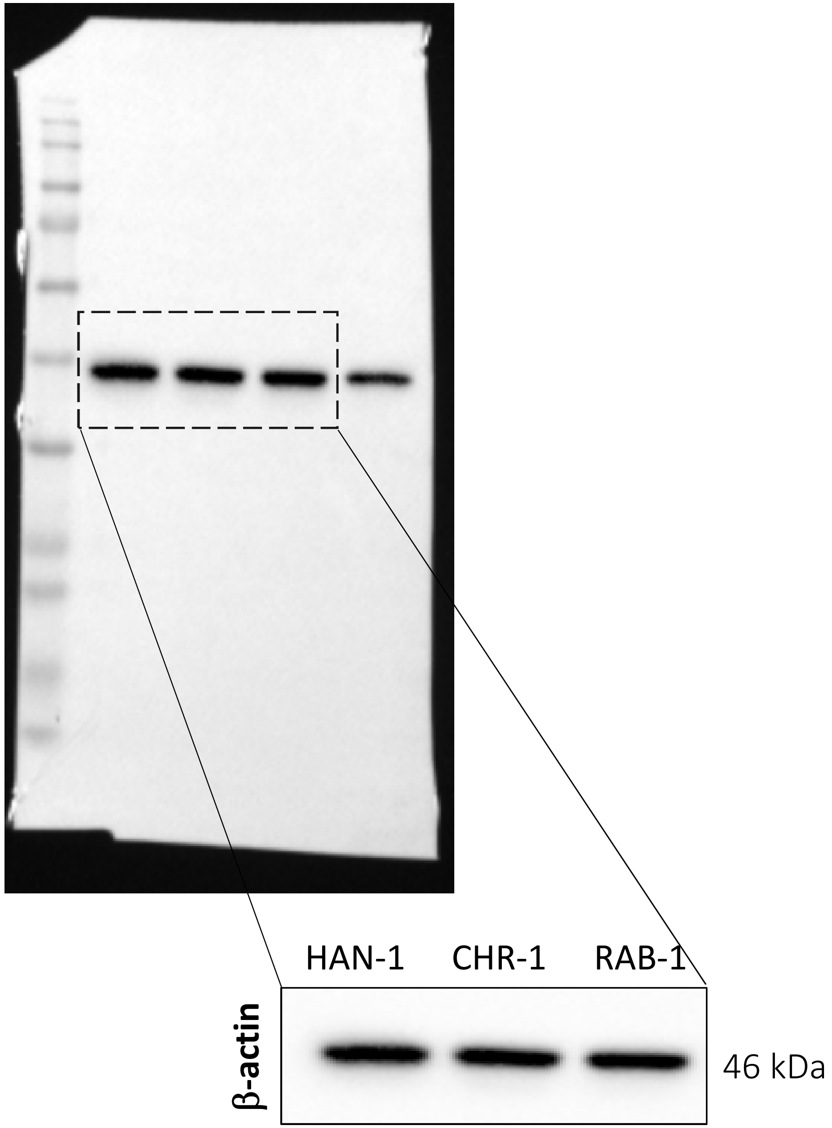

β-actin
